# The Dynamic Localization of Cytoplasmic Dynein in Neurons Is Driven by Kinesin-1

**DOI:** 10.1016/j.neuron.2016.04.046

**Published:** 2016-06-01

**Authors:** Alison E. Twelvetrees, Stefano Pernigo, Anneri Sanger, Pedro Guedes-Dias, Giampietro Schiavo, Roberto A. Steiner, Mark P. Dodding, Erika L.F. Holzbaur

**Affiliations:** 1 Department of Physiology, Perelman School of Medicine, University of Pennsylvania, Philadelphia, PA 19104-6085, USA; 2 Molecular NeuroPathobiology Laboratory, Sobell Department of Motor Neuroscience & Movement Disorders, UCL Institute of Neurology, University College London, London, WC1N 3BG, UK; 3 Randall Division of Cell and Molecular Biophysics, King’s College London, London, SE1 1UL, UK

## Abstract

Cytoplasmic dynein, the major motor driving retrograde axonal transport, must be actively localized to axon terminals. This localization is critical as dynein powers essential retrograde trafficking events required for neuronal survival, such as neurotrophic signaling. Here, we demonstrate that the outward transport of dynein from soma to axon terminal is driven by direct interactions with the anterograde motor kinesin-1. In developing neurons, we find that dynein dynamically cycles between neurites, following kinesin-1 and accumulating in the nascent axon coincident with axon specification. In established axons, dynein is constantly transported down the axon at slow axonal transport speeds; inhibition of the kinesin-1-dynein interaction effectively blocks this process. In vitro and live-imaging assays to investigate the underlying mechanism lead us to propose a new model for the slow axonal transport of cytosolic cargos, based on short-lived direct interactions of cargo with a highly processive anterograde motor.

**Video Abstract:**

## Highlights

• Dynein localization is dynamic prior to axon specification
• Dynein is enriched distally in axons via slow axonal transport
• Anterograde trafficking of dynein is driven by a direct interaction with kinesin-1
• Slow transport of cytosolic cargos involves transient links to processive motors

## Teaser

Twelvetrees et al. examine dynein localization in neurons and find that dynein dynamically relocalizes among neurites prior to axon specification. Following specification, dynein is constitutively trafficked down the axon via transient, but direct, interactions with the processive motor kinesin-1.

## Introduction

In neurons most pre-synaptic proteins are synthesized in the soma and are then transported long distances to reach their site of action. The continuous synthesis and delivery of new axonal proteins is essential to neuronal function (Kleim et al., 2003). In vivo pulse chase labeling experiments identified two major forms of anterograde axonal transport (see Roy, 2014 for recent review): a fast component (FC) associated with vesicular organelles moving between 50–200 mm/day and a slow component of cytoskeletal and cytoplasmic proteins moving 0.2–10 mm/day, which can be further subdivided into slow component a (SCa) and slow component b (SCb). The transit of new axonal and synaptic constituents via slow transport can take up to a year for cells with extended axons such as human motor neurons. However, at least three times the amount of protein is delivered to pre-synapses by slow compared to fast transport (Garner and Mahler, 1987), making this the major protein delivery system.

There has been significant progress in our understanding of fast axonal transport (Maday et al., 2014), but slow axonal transport has been more difficult to study. Groundbreaking work on neurofilament transport demonstrated that while overall rates of slow transport are orders of magnitude less than fast transport, there are no slow transport-specific motor proteins (Brown et al., 2005, Roy et al., 2000, Wang et al., 2000); instead, slow transport relies on the same microtubule motors that mediate fast vesicular transport (He et al., 2005, Wagner et al., 2004). Consequently, instantaneous velocities for neurofilaments are similar to those measured for vesicular cargos, but overall transport rates are dominated by long off-track times in a “stop and go” model for transport (Brown et al., 2005).

The slow transport of diffuse cytosolic proteins of SCb has been even more difficult to study, as there is no discrete unit to track through time. Recent success has been achieved using photoactivation to monitor SCb proteins synapsin and CaMKII (Scott et al., 2011). This work led to the proposal that presynaptic proteins transiently aggregate and associate with passing vesicles in a “dynamic-recruitment” model (Scott et al., 2011, Tang et al., 2013). However, the molecular mechanisms driving these dynamics remain undefined.

Here, we focus on the anterograde axonal transport of cytoplasmic dynein. Metabolic labeling studies indicate that ∼80% of newly synthesized dynein moves outward as a component of slow axonal transport at SCb velocities (1–10 mm/day) (Dillman et al., 1996a, Dillman et al., 1996b). Cytoplasmic dynein is the major retrograde motor in axons, yet it cannot drive its own localization to the axon terminal because the uniform microtubule polarity in axons directs active dynein back to the soma. Dynein is essential for many axonal functions including: growth cone extension (Grabham et al., 2007, Myers et al., 2006); axon elongation (Roossien et al., 2014); retrograde neurotrophic signaling (Heerssen et al., 2004, Yano et al., 2001); and autophagy (Maday et al., 2012). Consequently, mutations in dynein lead to neurodevelopmental and neurodegenerative phenotypes in both humans and mice (Schiavo et al., 2013). Although the distal localization of dynein in the axon is essential to normal neuronal function, how this is achieved is unknown.

**Figure 1:**
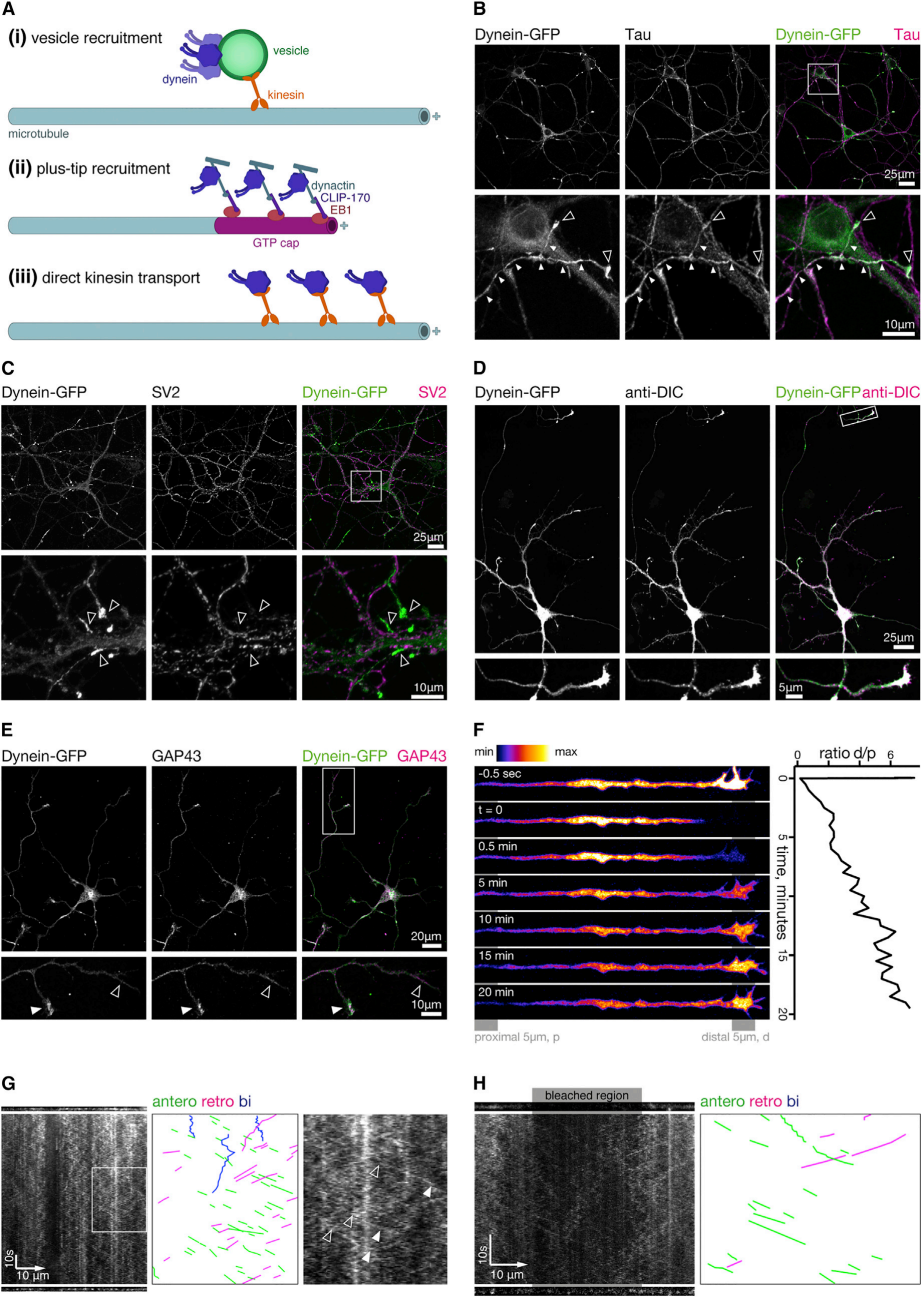
Dynein-GFP Accumulates in the Axon Terminals of Primary Hippocampal Neurons throughout Development (A) Models for dynein transport in the axon: (i) carrying extra dynein motors on vesicles; (ii) recruitment and surfing of microtubule +TIPs; and (iii) direct transport by kinesin. (B–E) Immunofluorescence and confocal microscopy of dynein-GFP primary hippocampal neurons. (B) Dynein-GFP accumulates in tau-positive axons at 8 DIV (small arrowheads), with particular enrichment in the axon terminals (empty arrowheads). Dynein-GFP accumulation does not colocalize with the presynaptic marker SV2 at 8 DIV (Figure S1A) or 21 DIV (C, empty arrowheads), or with the postsynaptic marker PSD-95 (Figure S1B). (D) Dynein-GFP is colocalized with anti-DIC (colocalization appears white, neuron shown at 12 DIV). (E) Dynein-GFP is colocalized with GAP43 at 3 DIV (filled arrowhead, GAP43-positive terminal; empty arrowhead, GAP43-negative terminal). (F) FRAP analysis of dynein-GFP localization to axon terminals shows recovery of distally accumulated dynein takes >20 min (left). Quantification (right) shows ratio of the mean intensity of the distal 5 μm, d, over the more proximal 5 μm region, p, 50 μm away. (G) Left: first frame, kymograph, and last frame of a dynein-GFP hippocampal axon imaged using near-TIRF. Scale bar indicates anterograde direction. Middle: selected events highlighted for clarity. Right: enlargement of boxed region. Arrowheads point to start (empty) and end (filled) of selected anterograde events. (H) Left: first frame, kymograph and last frame of a dynein-GFP hippocampal axon imaged by near-TIRF after bleaching. Scale bar indicates anterograde direction. Right: selected events highlighted for clarity.

There are three mechanisms that could describe the anterograde transport of dynein on microtubules (Figure 1A): (1) transport of dynein on vesicles, (2) “surfing” of dynein on polymerizing microtubule plus ends, and (3) direct transport by kinesin. While a small fraction of dynein motors are trafficked down the axon on fast-moving vesicles (Dillman et al., 1996a, Dillman et al., 1996b), this population of motors is stably attached to organelles (Encalada et al., 2011, Hendricks et al., 2010) and unavailable for other essential dynein-driven functions. Alternatively, anterograde transport of dynein could result from dynein recruitment to growing microtubule plus ends via binding to the plus-end-tracking proteins (+TIPs) EB1 and CLIP-170 through dynactin (Moughamian et al., 2013, Nirschl et al., 2016). Due to their uniform polarity, polymerizing microtubules within the axon produce +TIP comets that move away from the soma; dynein could potentially “surf” the +TIP wave toward the axon tip. Finally, in support of the direct recruitment model, a minimal complex for dynein transport by kinesin was recently reconstituted from yeast proteins (Roberts et al., 2014), indicating that this scenario is also possible.

To test these possibilities, we used a dynein-GFP mouse: a knockin model with tandem GFP and FLAG tags fused in frame to the C terminus of the neuron-specific isoform of the dynein intermediate chain (DIC1) (Zhang et al., 2013). Thus, tagged DIC1 is expressed at endogenous levels and incorporates into the multi-subunit dynein complex to produce functional motors (Ayloo et al., 2014, Zhang et al., 2013). We used live cell imaging at different spatiotemporal scales to define the anterograde movement of the dynein-GFP population, in combination with in vitro biochemistry and single molecule assays to identify a motile dynein:kinesin complex. These studies provide new mechanistic insight into the active transport and localization of an essential neuronal motor by demonstrating a robust kinesin-dependent, but non-vesicular, translocation of dynein along the axon of primary hippocampal neurons.

## Results

### Dynein Preferentially Accumulates in Distal Axons throughout Development

As an important first step, we wanted to characterize the distribution of cytoplasmic dynein in our model system of primary hippocampal neurons isolated from the dynein-GFP knockin mouse. In hippocampal neurons at 8 days in vitro (DIV), dynein-GFP was present throughout the somatodendritic and axonal compartments. However, the distribution was not homogeneous as there was a striking accumulation of dynein-GFP in the distal ends of axons, appearing as many bright puncta in larger fields of view (Figures 1B–1D, top panels). Axonal accumulation of dynein-GFP was confirmed by co-staining neurons with antibodies to GFP and the axonal marker tau. While not co-localized, both proteins were consistently enriched within the same cellular compartment (Figure 1B, empty arrowheads indicate dynein in axon terminals). We did not observe co-localization of dynein-GFP puncta with the pre-synaptic marker SV2 either at 8 DIV (Figure S1A) or in established cultures at 21 DIV (Figure 1C). These observations suggest that dynein-GFP is preferentially localized to growth cones and the distal axon, which we confirmed by co-staining stage 3 (DIV3) dynein-GFP hippocampal neurons with anti-GFP and the early growth cone marker GAP43 (Figure 1E, filled arrowhead). Thus, dynein accumulates in the distal axon, far from its site of synthesis. This distal localization was not limited to the GFP-tagged neuronal isoform, as an anti-DIC antibody recognizing both tagged DIC1 and the ubiquitously expressed DIC2 isoform showed a near identical localization (Figure 1D, analysis of axon tips gives a mean Pearson’s coefficient of 0.80 ± 0.03; n = 18 from 2 independent cultures).

To understand the dynamics of dynein-GFP accumulation, we performed fluorescence recovery after photobleaching (FRAP) experiments on growth cones at 8 DIV (Figure 1F). We found that distal dynein-GFP accumulations recovered very slowly after bleaching, indicating that cytoplasmic dynein does not undergo free diffusion. However, by comparing the distal bleached 5 μm to a more proximal axon region, we found that although absolute fluorescence intensity did not recover within 20 min, the relative enrichment of dynein distally compared to more proximal regions of the axon did recover (Figure 1F).

In these FRAP experiments, it was difficult to visualize individual transport events driving signal recovery. To improve our signal to noise, we imaged axonal dynein-GFP using near-total internal reflection fluorescence microscopy (near-TIRF). Axon kymographs were generated (Figures 1G and 1H) with anterograde and retrograde events appearing as diagonal lines angled right and left, respectively. These kymographs showed many short anterograde events of comparatively uniform velocity and low signal intensity. When we bleached regions of the axon and imaged recovery with near-TIRF, the same uniform velocity events were observed to move into the bleached area (Figure 1H). Typical anterograde events had a speed of ∼1 μm/s, equivalent to the known speed of kinesin-1.

### Dynein Accumulates in Neurites Coincident with Axon Specification

As dynein-GFP is already accumulated in the growth cones of young (stage 3) neurons, we wondered whether dynein accumulated in the nascent axonal neurite prior to, or only after, axon specification. To answer this question, we carried out overnight live cell imaging of axon specification in developing stage 2 hippocampal neurons from dynein-GFP mice. Stage 2 development is characterized by the protrusion of several short neurites with plus-end out microtubule orientation (Baas et al., 1989). Upon axon specification in stage 3, one neurite undergoes a period of growth, simultaneously acquiring an axonal phenotype (Jacobson et al., 2006).

**Figure 2:**
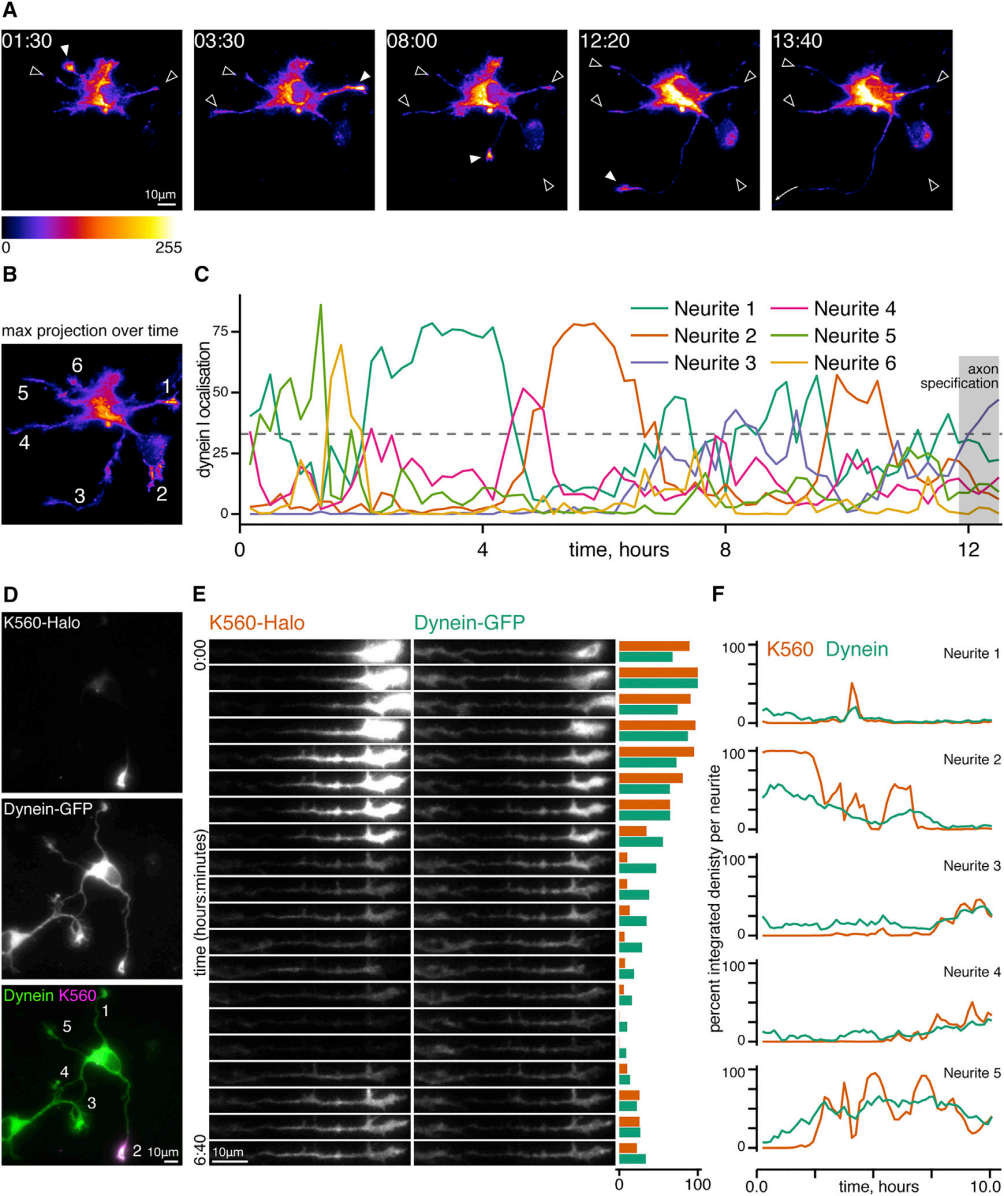
Dynein Accumulates in the Growth Cone Coincident with Axon Specification (A) Still images from Movie S1 showing neurite outgrowth in dynein-GFP stage 2 neuron. Filled arrowhead, neurite with highest dynein-GFP intensity; empty arrowheads, other neurites. Long arrow shows direction of axon exit. Time stamps, hours:minutes; fluorescence intensity scale, bottom left. (B) Maximum projection of Movie S1 with neurite labeling used in (C). (C) Individual neurite integrated density as a percentage of the total in all neurites from Movie S1 through time. Neurites labeled as in (B). (D) Maximum projection of Movie S2, dynein-GFP neuron transfected with K560-Halo, indicating neurite labeling for (E) and (F). (E) Still images of Neurite 2 (see D) through time. Quantification bars show relative integrated density of K560 (orange) and dynein-GFP (green) within Neurite 2 over time. (F) Individual neurite integrated density as a percentage of the total in all neurites from Movie S2 through time. Neurites labeled as in (D). See also Figure S2.

To our surprise, prior to axon specification the accumulation of dynein-GFP in any one neurite was highly dynamic and transient, with accumulation apparent in only one or two neurites at any given time (Figure 2A and Movie S1; filled arrow head indicates dynein accumulation). This behavior could be quantified by expressing the integrated density of dynein-GFP fluorescence in one neurite as a percentage of the total dynein-GFP signal in all neurites (Figures 2B and 2C). Transient accumulations of dynein moving from neurite to neurite were seen right up until the moment of axon specification, when dynein became enriched in the nascent axonal neurite coincident with the characteristic growth spurt (Figure 2C). Dynein accumulation persisted during extension of the axonal growth cone (Figure 2C and Movie S1). Although some dynein-GFP remains in the somato-dendritic compartment throughout development, we did not observe any further prominent accumulations in young dendrites during this time frame.

The behavior of dynein-GFP in this assay was strikingly reminiscent of observations on the transient and stochastic oscillation of the constitutively active tailless kinesin-1 construct, K560, among developing neurites (Jacobson et al., 2006). These oscillations are followed by the stable accumulation of K560 in a single neurite as one of the earliest markers of axonal identity, leading to the hypothesis that this selectivity ensures that kinesin-1 cargoes are targeted to just one neurite. Thus, we compared the dynamics of dynein and kinesin by expressing Halo-tagged K560 labeled with tetramethylrhodamine (TMR) in hippocampal neurons from the dynein-GFP mouse. Dynein-GFP and K560-Halo can be seen co-migrating among stage 2 neurites prior to axon specification (Movie S2 and Figures 2D and 2E). The co-migration of these two markers was not an artifact due to volume changes, as the cytosolic marker BFP did not show the same behavior (Figure S2 and Movie S3). When the integrated density of neurite fluorescence was quantified (Figure 2F), the movement of K560 among neurites was found to be faster and more complete than dynein-GFP, resulting in sharper, more defined peaks over time, although the same overall dynamics were observed. This difference likely reflects the fact that K560, lacking key regulatory domains and unbound from cargo, is free to rapidly respond to changes in the microtubule cytoskeleton, whereas dynein-GFP is part of an endogenous complex with multiple cellular roles to perform.

### Dynein Intermediate Chain Interacts Directly with Kinesin-1

The co-migration of dynein-GFP and K560 in developing neurites suggests that there may be a kinesin-1-dependent mechanism for the anterograde transport of dynein in the axon. Previous work from our lab identified a direct interaction between the neuron-specific DIC1 isoform of dynein and kinesin-1 (Ligon et al., 2004), raising the possibility that dynein complexes are directly transported by kinesin-1 in the axon. We set out to characterize the dynein-kinesin interaction in more detail to examine this possibility.

**Figure 3:**
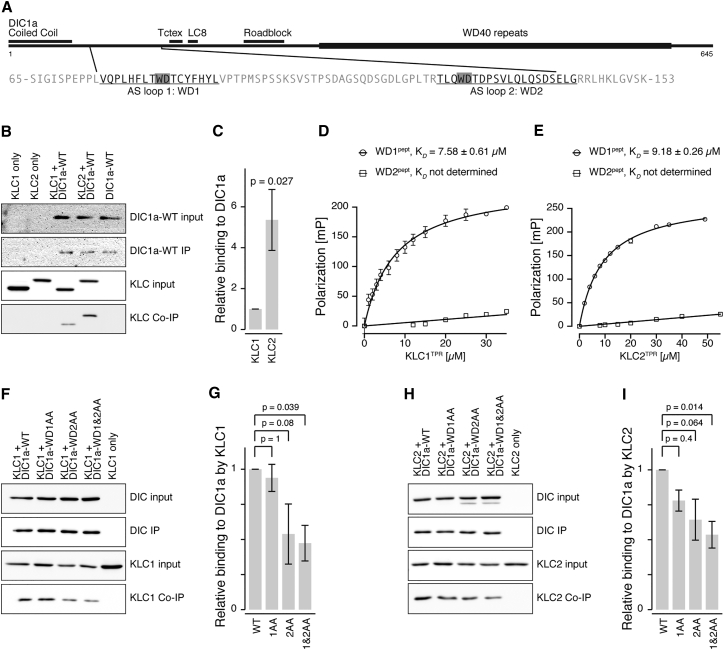
DIC1 Interacts Directly with KLCs through DIC1 Tryptophan Motif Binding to the KLC TPR Domains (A) Schematic of DIC1 showing structural motifs and dynein light chain binding sites relative to the alternatively spliced regions (AS loops 1 and 2) and WD motifs (WD1 and WD2, in gray). See also Figure S3. (B and C) COS cells cotransfected with mCherry-tagged DIC1a and HA-tagged KLC as indicated followed by immunoprecipitation (IP) with anti-mCherry (B, western blotting with anti-DIC and anti-HA). (C) Co-IP efficiency expressed as band intensity relative to KLC1 ± SEM; n = 4 experiments. (D and E) Fluorescence polarization measurements with peptides of the first and second DIC1 tryptophan motifs (WD1^pept^ and WD2^pept^, respectively) binding to the TPR domain of KLC1 (D) and KLC2 (E). K_D_ values determined at 150 mM NaCl; error bars ± SEM, experiments typically done in triplicate. See also Figure S3. (F–I) COS cells cotransfected with mCherry-tagged DIC1a (wild-type, WT; or point mutations of WD1, WD2, or both WD1 and 2 to alanine, AA) and HA-tagged KLC1 (F) or KLC2 (H) as indicated followed by IP with anti-mCherry (F and H, western blotting with anti-DIC or anti-HA). (G and I) Co-IP efficiency expressed as band intensity relative to WT DIC1a ± SEM; n = 6 and 4 experiments, respectively.

Endogenous kinesin-1 is a tetramer formed of two heavy chains (encoded by KIF5A, KIFB, or KIF5C) and two light chains (KLC1 and KLC2). We previously identified an interaction between DIC1 and KLC, with a strong preference for KLC2 over KLC1 in in vitro binding assays (Ligon et al., 2004). We confirmed these previous studies by overexpressing full-length proteins from COS cell lysates (Figure 3B) and found that 5-fold more KLC2 than KLC1 was co-immunoprecipitated with DIC1a in this assay (Figure 3C).

Tryptophan-based motifs mediate binding to the tetratricopeptide repeat (TPR) domains of KLCs (Dodding et al., 2011, Pernigo et al., 2013). DIC1 was identified in a bioinformatics search using a bipartite tryptophan motif consensus sequence (Dodding et al., 2011), and the location matched our previously reported KLC binding site on DIC1 (Ligon et al., 2004) (Figure 3A). These motifs (WD1 and WD2) lie within two short alternatively spliced sequences in DIC1 (AS loop 1 and AS loop 2, respectively, see Figure 3A and Figure S3) but are absent from the ubiquitously expressed isoform DIC2c (Kuta et al., 2010), thus only neuronal dynein is predicted to bind KLC using these motifs.

To probe the role of the tryptophan motifs, we tested the ability of peptides derived from the AS1 and AS2 loops to interact with purified KLC1 or KLC2 TPR domains using fluorescence polarization (Figures 3D and 3E). A TAMRA-conjugated 11-amino-acid-long peptide centered on the first tryptophan motif (WD1^pept^) bound to KLC1^TPR^ with a K_D_ of 7.58 ± 0.61 μM and to KLC2^TPR^ with a K_D_ of 9.18 ± 0.26 μM. These affinities are similar to those of peptides with tryptophan motifs from either the lysosomal cargo adaptor, SifA-kinesin interacting protein (SKIP), or Calsyntenin-1 (Pernigo et al., 2013). In contrast, a peptide centered on the second tryptophan motif (WD2^pept^) did not show an interaction in this assay, similar to the behavior of the second tryptophan motif within the SKIP protein (Pernigo et al., 2013). WD1^pept^ polarization was reduced in a concentration-dependent manner by titrating increasing amounts of unlabeled SKIP peptides, consistent with their binding being mutually exclusive, as they likely occupy the same topological location on KLC2^TPR^ (Figure S3D).

To examine the contribution of the tryptophan motifs to the DIC-KLC interaction in the context of full-length proteins, we tested the effect of alanine substitutions of either the tryptophan or the following aspartic acid residues of WD1 and WD2 on the ability of DIC1 to co-immunoprecipitate KLCs (Figures 3F–3I). Three mutants were examined: alanine mutation of WD1 (WD1AA), WD2 (WD2AA), and WD1 and 2 combined (WD1 and 2AA). For both KLC1 (Figures 3F and 3G) and KLC2 (Figures 3H and 3I), only the double mutant of both WD1 and 2 showed a consistent decrease in relative binding to KLC. Thus, although binding between the WD2 peptide and KLC^TPR^ was not detected in vitro, both motifs are important in the context of the full-length proteins.

**Figure 4:**
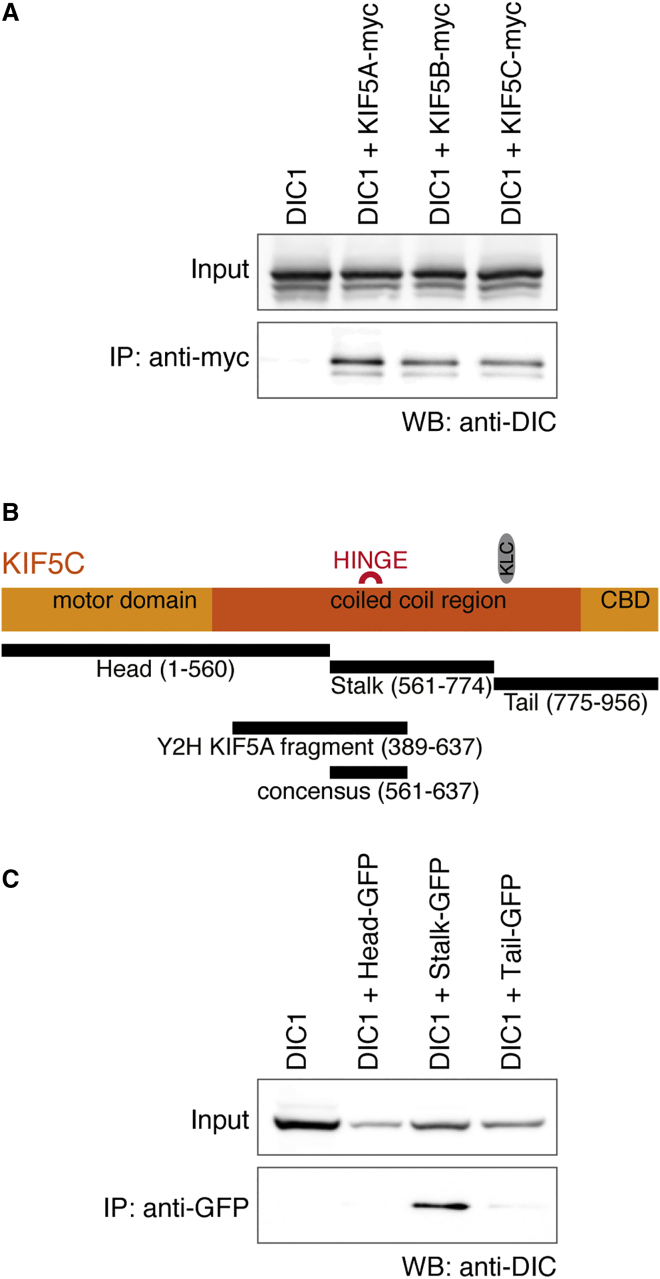
DIC1a Interacts with the Central Stalk Region of KIF5 Heavy Chains (A) Western blot (WB) of COS cells cotransfected with mCherry-tagged DIC1a and myc-tagged KIF5A-C constructs as indicated followed by immunoprecipitation with anti-myc. (B) Schematic of KIF5 showing the constructs used in (C), the sequence isolated by yeast two-hybrid screen and the resulting consensus region for binding DIC relative to key domains: motor domain, coiled-coil, cargo binding domain (CBD), central hinge, and KLC binding region. (C) Western blot (WB) of COS cells cotransfected with mCherry-tagged DIC1a and GFP-tagged KIF5C “head,” “stalk,” and “tail” constructs shown in (B) followed by immunoprecipitation with anti-GFP.

An emerging commonality of active kinesin-1 transport complexes is the formation of multiple interactions between the motor and the cargo, including associations with both KLCs and the KIF5 heavy chain (KHC). For example, the cargo adaptor proteins HAP1, JIP1, and JIP3 all interact with both KIF5 and KLC (reviewed in Fu and Holzbaur, 2014). Consistent with this model, in a yeast two-hybrid screen for binding partners of DIC1a (Perlson et al., 2013), we identified residues 389–637 of KIF5A, a region conserved among all three KIF5 isoforms (57.4% and 62.2% identity with KIF5B and KIF5C, respectively). Consistently, immunoprecipitations with all three myc-tagged KIF5 isoforms co-precipitated DIC1a (Figure 4A). We further mapped the DIC1a interaction domain in immunoprecipitation experiments with KIF5C “head” (1–560), “stalk” (561–774), and “tail” (775–956) constructs. DIC1a was co-precipitated with the stalk region of KIF5C, identifying a consensus binding region-spanning residues 561–636 of KIF5C (Figures 4B and 4C). This region encompasses the central hinge region of KIF5 that allows the motor to fold to form an auto-inhibited conformation (Friedman and Vale, 1999, Hackney et al., 1992). Effector binding to this hinge region can stabilize the open conformation of kinesin-1 to create an active transport complex (Fu and Holzbaur, 2013).

In summary, interaction between DIC and kinesin-1 is mediated by neuron-specific isoforms of DIC, KLC TPR domains, and the stalk region of the KIF5 heavy chains. Together, these interactions are likely to stabilize a dynein-kinesin complex capable of active transport.

### An Endogenous Complex of Kinesin-1 and Cytoplasmic Dynein Is Found in Brain

Next, we asked whether dynein-kinesin complexes form in the brain. Cytosolic dynein can be enriched from a 100,000 × *g* supernatant fraction from mammalian brain (Ayloo et al., 2014); dynein from this fraction is readily separated from kinesin using a sucrose gradient (see Figure S4A) and so an endogenous dynein:kinesin complex is unlikely to be found in this fraction. Instead, Scott et al. proposed that after differential centrifugation of homogenized brain, the synaptosome-depleted (S2) fraction could be reasonably assumed to contain the majority of material that is moved within axons (Scott et al., 2011), including soluble cytosolic proteins, small vesicles, and macromolecular protein complexes. These three phases can be further separated into a soluble cytosolic protein fraction (S3) and the remaining pellet (P3) with a 100,000 × *g* spin. Resuspension of P3 and bottom loading onto a sucrose gradient yields floating small vesicles (V) separated from macro protein complexes (PC), which remain within high-density fractions at the bottom of the gradient. Following this procedure, Scott and colleagues found that a substantial fraction of the cytosolic proteins CaMKII and synapsin, known to travel by slow axonal transport, were found in association with both floating vesicles and the large protein complexes at the bottom of the gradient. Based on these observations, we sought to isolate an endogenous non-vesicular dynein:kinesin complex that may be responsible for the slow transport of dynein.

**Figure 5:**
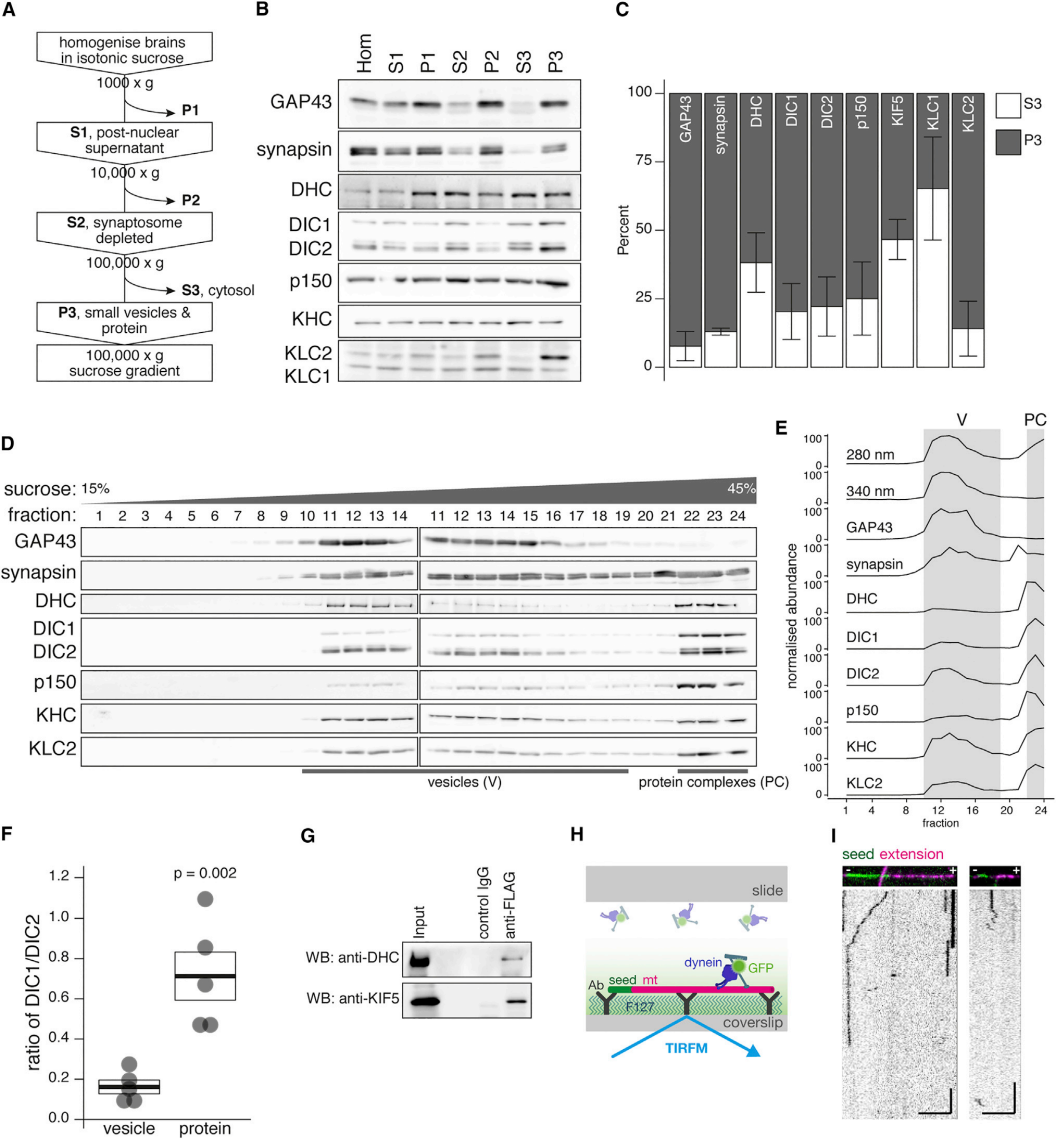
Biochemical Analysis of Endogenous Dynein-GFP and Kinesin Complexes from Brain (A) Experimental procedure for dynein-GFP mouse brain differential centrifugation. (B) Distribution of proteins of interest across centrifugation steps outlined in (A) by SDS-PAGE and western blotting. Antibodies for: vesicular marker GAP-43; slow transport marker synapsin; and motor protein subunits DHC, DIC, p150, KHC, and KLC. (C) Quantification of the relative abundance of each protein in fractions S3 versus P3. n = 3; error bars ± SEM. (D) Distribution of proteins of interest across the 24 fractions of the P3 sucrose density gradient, showing separation of vesicles (V, fractions 10–19) from high-density protein complexes (PC, fractions 22–24). Protein association with vesicles was attenuated by the addition of Triton X-100 to the resuspended P3 fraction (Figure S4). Fractions 11–14 were loaded in duplicate to normalize band intensities across the two gels required to run all fractions. (E) Quantification of the gradient assay in (D) highlighting the vesicular (V) and high-density protein fractions (PC). (F) The ratio of DIC1 to DIC2 in vesicle fractions from the gradient assay compared to high-density protein fractions. n = 5 (gray circles), mean ratio (heavy line) ± SEM (box) is shown. (G) KIF5 and DHC co-immunoprecipitate in an endogenous complex from fractions 23 and 24 (D) with anti-FLAG. (H) Schematic of experimental set up for (I). Rhodamine-labeled microtubules were extended from AMCA-labeled microtubule seeds to indicate polarity, then immobilized onto silanized coverslips by anti-tubulin antibody. Dynein-GFP events were imaged by TIRF microscopy (TIRFM). (I) Two polarity-marked microtubules and example kymographs of dynein-GFP events on those microtubules imaged by TIRFM. Vertical scale is 10 s; horizontal scale is 5 μm.

Brain homogenate from dynein-GFP mice was fractionated following the scheme in Figure 5A. Samples from fractions S1-P3 were adjusted to 1 μg/μL prior to SDS-PAGE and western blotting (Figure 5B). We compared the distribution of proteins between the cytosolic fraction (S3) and the P3 fraction that is enriched in small vesicles and macromolecules (Figure 5C). As expected, the vesicular marker GAP43 was largely concentrated in the vesicle-containing P3 pellet, as was the slow axonal protein synapsin. All dynein/dynactin subunits assessed had a similar distribution between the cytosolic S3 pool versus the vesicular/protein P3 pool (DHC, 38.2% ± 10.95%; DIC1, 20.4% ± 10.2%; DIC2, 22.2% ± 10.8%; p150, 25.1% ± 13.4% present in S3). The kinesin-1 heavy chain, KIF5, had a slightly higher proportion present in the cytosol than dynein/dynactin (46.6% ± 7.3% in S3 versus a mean of 26.5% for all dynein/dynactin subunits); however, a key difference was in the relative distributions of KLC1 and KLC2. 85.9% ± 10.0% of KLC2 was found in P3, compared to only 34.7% ± 18.8% of KLC1. The relative enrichment of KLC2 in P3 suggests a key functional difference between KLC1 and KLC2 in brain.

To separate vesicles from protein complexes within P3, we bottom loaded the resuspended pellet onto a 15%–45% sucrose gradient so that vesicles could be separated by their lower density (Figures 5D and 5E). Equal volumes of the 24 gradient fractions were used for SDS-PAGE and western blotting (Figure 5D). Protein concentrations from each fraction were assessed by absorbance at 280 nm and the vesicle distribution through the fractions was assessed by turbidity at 340 nm (Figure 5E). The vesicle marker GAP43 showed clear separation from the bottom of the gradient and good agreement with the turbidity measurement of the samples, indicating that the majority of vesicles floated between fractions 10 to 19 (marked as “V” in Figure 5E). Measurements of absorbance at 280 nm showed a sharp rise in protein concentration between fractions 21 and 22; thus, fractions 22–24 were designated as the high-density protein fractions (“PC” in Figure 5E). There was relatively more protein associated with the “V” fractions compared to “PC” fractions. All motor subunits assessed had distinct vesicle- and protein-associated pools, and in fact showed a substantial enrichment in the densest fractions relative to the total protein distribution. An intriguing difference was in the distribution of DIC isoforms 1 and 2 between vesicles and dense protein fractions. In the dynein-GFP mice, DIC1 is separated from DIC2 by the molecular weight of the exogenous GFP tag; using the anti-DIC antibody to detect both isoforms we directly compared the ratio of DIC1 to DIC2 from band intensities (Figure 5F). The ratio of DIC1/DIC2 in the vesicular fraction was 0.16 ± 0.03, whereas in the protein fraction the DIC1/DIC2 ratio was 0.71 ± 0.1, a 4.4-fold increase. This finding demonstrates isoform specificity between the pools of dynein and is consistent with the relative enrichment of both DIC1 and KLC2 in the PC fraction.

To isolate a possible non-vesicular dynein:kinesin complex, we carried out immunoprecipitation of dynein from the high-density fractions 23–24, using antibodies to the 3xFLAG tag knocked into the DIC1 C terminus. Using this strategy, we co-precipitated dynein heavy chain and kinesin heavy chain using anti-FLAG antibody (Figure 5G).

Next, we asked whether the protein in high-density fractions of the sucrose gradient was functional—that is, whether it represents native macromolecular complexes or alternatively is comprised of protein aggregates precipitated during fractionation. We analyzed the protein fraction by single molecule TIRF microscopy using polarity marked microtubules (Figure 5H). GFP puncta bound to microtubules (Figure 5I) and we observed both minus-end-directed (left) and plus-end-directed (right) motility. While the extent of binding and motility from prep to prep was highly variable, likely due to the dilute nature of the protein fractions, qualitatively the events observed differed from the motility of either purified dynein or dynein-associated vesicles, as fewer directional switches were observed (Hendricks et al., 2010, Ross et al., 2006). Plus-end-directed motility in this assay is consistent with the anterograde bias of slow transport observed in cells; the minus-end-directed motility that was also observed may represent partial activation of dynein activity under the conditions of the assay, for example, by loss through dilution of a negative inhibitor such as Lis1.

### Anterograde Bias in Dynein Transport Can Be Directly Observed

**Figure 6:**
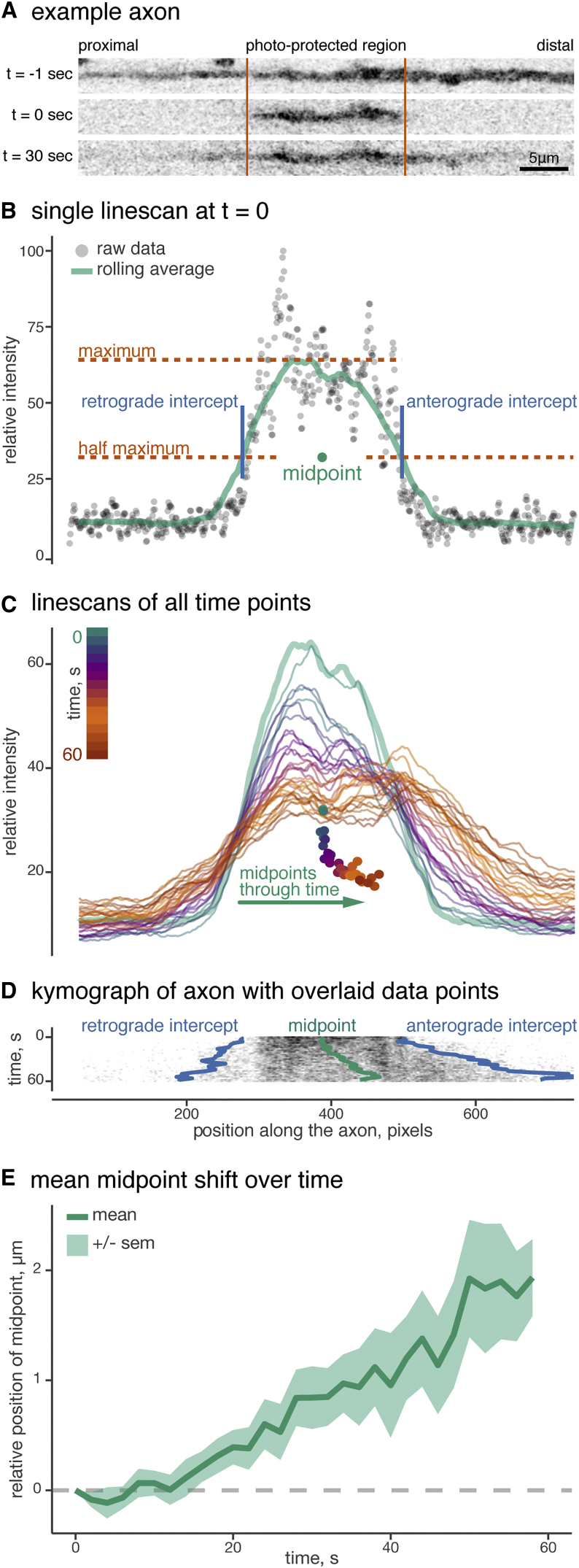
Discrete Populations of Dynein-GFP Move with an Anterograde Bias in the Axon (A) Example axon before (t = –1) and after (t = 0) bleaching. The relative position of the soma is indicated. (B) Midpoint calculation of dynein-GFP photo-protected region for t = 0. The relative intensity of the axon line scan at t = 0, showing raw data (gray dots) and rolling average (green line). Anterograde and retrograde fronts of the photo-protected population are defined as the positions along the axon where the intensity is half-maximal. The midpoint is defined as halfway between these two intercepts. (C) Line scans and midpoints for all time points of the movie, demonstrating a displacement toward the distal axon with time. Color changes with time indicated by the scale bar, top left. (D) Kymograph of dynein-GFP axon from (A) showing anterograde movement of the photo-protected region, over plotted with the calculated midpoints and intercepts from (C). (E) The mean displacement of the midpoint through time (green line) from n = 11 neurons, ±SEM (green ribbon).

As noted above, there is a persistent anterograde bias in dynein transport throughout development, leading to accumulation in the peripheral axon and growth cone (Figure 1). Such an accumulation, which is the reverse of what would be expected to result from a passive diffusion from the site of synthesis, requires active transport in the anterograde direction. Recent success in imaging slow axonal transport of cytosolic proteins used photoactivation to create a discrete pool of protein that could be tracked in real time (Scott et al., 2011). We modified this assay in two ways. First, we used photobleaching to establish a discrete photo-protected population of dynein-GFP, expressed at endogenous levels in neurons, flanked by bleached areas allowing us to monitor movement along the axon. Second, we developed a new quantification method to track the midpoint of the photo-protected pool through time (Figure 6).

Primary hippocampal neurons from dynein-GFP mice were cultured for 8–10 DIV in 900 μm microfluidic devices in order to image single axons of known polarity. Post bleach motility was imaged for 60 s at 0.5 fps. A line scan was made for every frame of the time series, then movement of the photo-protected dynein-GFP pool was quantified by defining retrograde and anterograde edges at the half-maximal fluorescence value and tracking the displacement of the midpoint (Figure 6B). The peak of fluorescence intensity decreased with time as the dynein-GFP pool shifted in both anterograde and retrograde directions. However, the relative midpoint could be seen moving with a clear bias in the anterograde direction for several seconds (Figure 6C), particularly when overlaid onto the more standard kymograph representation of axonal transport (Figure 6D). Plotting the mean ± SEM of several axonal dynein-GFP displacements reveals a net anterograde bias of ∼1–2 μm/min (Figure 6E). By fitting a linear regression to each relative midpoint position over time, we observed anterograde velocities ranging from 0.01 to 0.1 μm/s. However, ∼80% of events were in the 0.01 to 0.05 μm/s range or approximately 1–5 mm a day. All net anterograde velocities were consistent with known speeds of slow axonal transport of cytoplasmic dynein in vivo (Dillman et al., 1996a, Dillman et al., 1996b).

### Anterograde Transport of Dynein Is Dependent on Microtubules and Interaction with the KLC TPR Domain

So far, we have established that cytoplasmic dynein moves with an anterograde bias along axons of developing hippocampal neurons, resulting in a distal accumulation following axon specification. Further, a dynein-kinesin-1 complex can be biochemically isolated from a non-vesicular fraction of brain. To directly test whether dynein is transported down the axon by kinesin, we utilized our axonal transport photobleaching assay.

**Figure 7:**
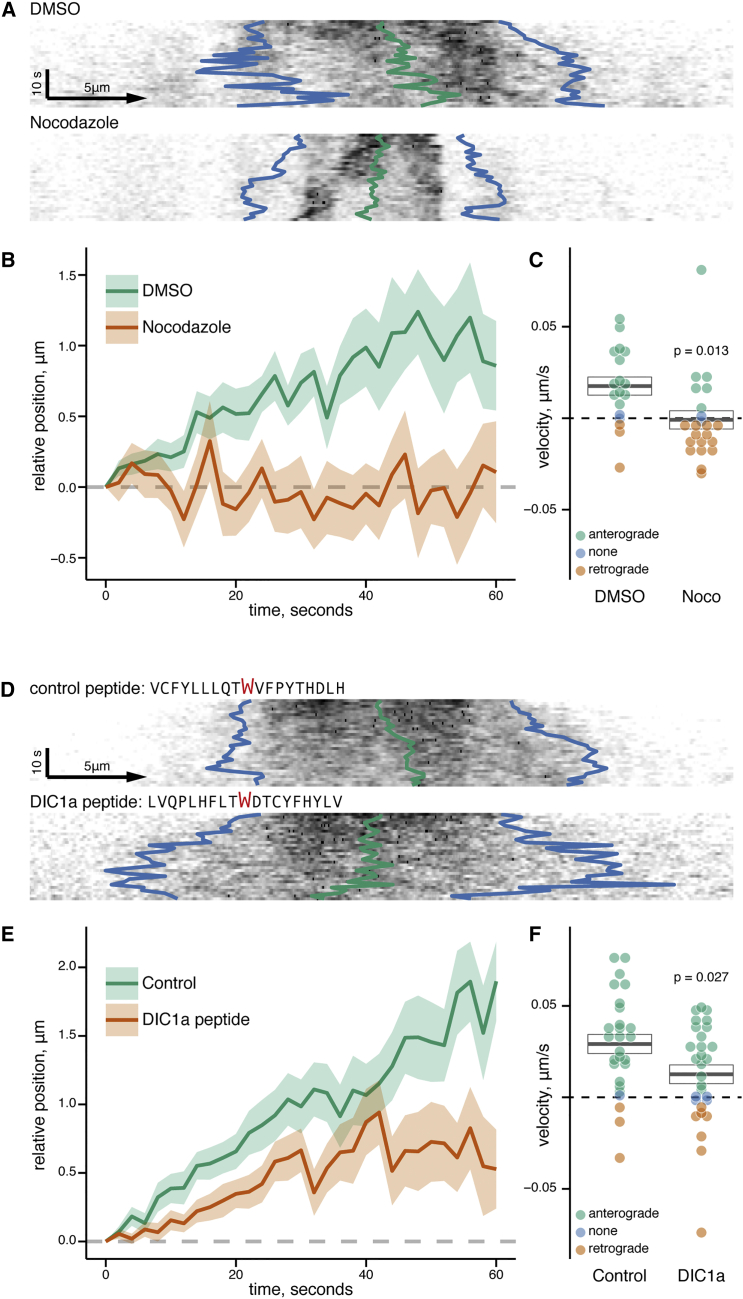
Slow Anterograde Transport of Dynein in the Axon Is Dependent on Microtubules and an Interaction with Kinesin (A) Kymographs of DMSO control and nocodazole-treated axons from dynein-GFP hippocampal neurons showing relative positions of anterograde and retrograde intercepts (blue) and the calculated midpoint displacement (green). Scale bar indicates anterograde direction. (B) The mean relative position of the midpoint with time for DMSO and nocodazole-treated axons: n = 18 and 22 axons, respectively, from three independent primary cultures; solid lines, mean; ribbons, ±SEM. (C) A linear regression was fitted to each axon’s midpoint displacement to find the velocity of displacement. The mean velocity (heavy line) ± SEM (box) is shown. Overlaid spots are the velocities for each measured kymograph with colors indicating overall direction of the kymograph. (D) Kymographs of control and DIC1a peptide-treated axons from dynein-GFP hippocampal neurons showing relative positions of anterograde and retrograde intercepts (blue) and the calculated midpoint displacement (green). (E) The mean relative position of the midpoint with time for control and DIC1a peptide-treated axons: n = 27 and 29 axons respectively from 3 independent primary cultures; solid lines, mean; ribbons, ±SEM. (F) Results of linear regression on each axon’s midpoint displacement to find the velocity of displacement. The mean velocity (heavy line) ± SEM (box) is shown. Overlaid spots are the velocities for each measured kymograph with colors indicating the overall direction of the kymograph.

First, we tested whether the anterograde transport of dynein was microtubule dependent, using nocodazole (final concentration 30 μM) to depolymerize microtubules. Every viable isolated axon (see Experimental Procedures) within the microgrooves of the chamber was imaged following the bleaching protocol described above. The relative position of the midpoint for each kymograph was calculated (see Figure 7A for examples) and the mean midpoint displacement ± SEM was plotted for all axons (Figure 7B). Nocodazole treatment abrogated the anterograde bias in transport seen in DMSO control neurons, with no net motility in either the anterograde or retrograde direction. To establish a mean velocity for dynein-GFP, we fitted each midpoint shift with a linear regression. In the DMSO control, dynein-GFP had a mean velocity of 0.018 ± 0.005 μm/s, whereas nocodazole treatment reduced this to –0.001 ± 0.005 μm/s (Figure 7C).

Axonal microtubules are uniformly orientated with polymerizing plus ends moving away from the soma. Dynein can be recruited to microtubule plus ends via dynactin and the +TIPs EB1 and CLIP-170 (Moughamian et al., 2013). Dynein surfing via +TIPs could be an alternative microtubule-dependent hypothesis for the anterograde transport of dynein in the axon (Figure 1A). We tested this by using taxol to stabilize microtubules and inhibit the formation of microtubule comets in the axon (Figure S5A). Taxol-treated axons showed no inhibition in the anterograde bias of dynein transport (Figures S5B–S5D). Of note, EB3 comets had a speed of 0.113 ± 0.002 μm/s (n = 399, ±SEM), which is ∼10-fold slower than the anterograde dynein events that we observed by near-TIRF imaging (Figures 1G and 1H).

To directly test our hypothesis that interactions between dynein and kinesin-1 are required for the anterograde transport of dynein in the axon, we designed a peptide to block the interaction between DIC1 and KLC. We chose a 19-amino-acid-long sequence centered on the tryptophan in WD1 of DIC1a. This incorporated the alternatively spliced region AS loop 1, the region that we demonstrated interacted directly with the TPR of KLC using in vitro binding assays (Figures 3D and 3E). The control peptide also had a central tryptophan residue, but the upstream and downstream sequences were scrambled, maintaining overall charge and residue composition (Figure 7D). Hippocampal neuronal cultures were pre-treated for 45–60 min with either DIC1a or control peptide, complexed with Chariot reagent for delivery across the plasma membrane. Using our photobleaching protocol followed by analysis of midpoint displacement through time for each kymograph, we found that DIC1-peptide-treated axons had a substantial reduction in the anterograde bias of dynein-GFP transport (Figure 7E). All dynein-GFP population velocities calculated by linear regression of individual midpoint shifts are shown in Figure 7F. In the presence of the control peptide, dynein-GFP had a mean velocity of 0.029 ± 0.005 μm/s, whereas the presence of DIC1a peptide reduced this by over 50% to 0.012 ± 0.005 μm/s. This reduction in mean velocity was accompanied by a more than doubling of the number of kymographs with no net direction bias or retrograde bias in the presence of DIC1a peptide (38% showed no or retrograde bias in DIC1a peptide experiments versus 15% in control experiments), as well as a complete loss of kymographs showing anterograde velocities over 0.05 μm/s (22% of control kymographs had a velocity over 0.05 μm/s; mean velocity of anterograde only kymographs was 0.036 ± 0.004 μm/s and 0.029 ± 0.003 μm/s for control and DIC1a peptides, respectively). Consequently, the anterograde transport of dynein-GFP in the axon is critically dependent on the interaction between dynein subunit DIC1a and the KLCs of kinesin-1.

## Discussion

Slow axonal transport is a well-established phenomenon in vivo thanks to extensive pulse chase labeling experiments in vertebrate nerves since the 1960s (reviewed recently in Roy, 2014). However, the underlying mechanisms driving this bulk transport phenomenon, and in particular the transport of non-cytoskeletal elements, are not well understood.

We have identified a non-vesicular complex of kinesin and dynein in the brain, which we show is responsible for the anterograde bias of dynein transport in the axon. This anterograde transport produces a net population velocity equivalent to those measured for SCb proteins in vivo. The complex is formed by direct interactions between the dynein intermediate chain and kinesin-1 heavy and light chains. Crucially, the KLC interaction is only seen with the neuron-specific isoform of DIC, DIC1, which preferentially binds KLC2 over KLC1 via paired WD motifs. Cellular fractionation experiments indicate that KLC2 is enriched in the high-speed pellet (P3) relative to KLC1, and DIC1 is enriched in non-vesicular sucrose fractions relative to DIC2. This demonstrates that dynein and kinesin are tailored to specific functions by their complement of accessory proteins. From our biochemical analysis, the majority of dynein is not associated with vesicles, which fits well with in vivo data on the axonal transport of dynein showing that 80% moves by slow axonal transport in the SCb fraction, with just 15% associated with vesicles (Dillman et al., 1996b).

A key difference between our study and previous radio labeling studies (Dillman et al., 1996a, Dillman et al., 1996b) is that our slow transport imaging analysis captures the total pool of labeled dynein, not just newly synthesized proteins. Observations on this total pool of dynein indicate that the flux of anterograde slow transport exceeds the opposing flux resulting from dynein-mediated retrograde trafficking events, thus leading to a net accumulation of dynein in the distal axon far from the site of synthesis (Figure 1) and essentially reversing the concentration gradient that would result by diffusion. While the relative balance of anterograde and retrograde trafficking may potentially shift in adults, producing slower transport rates and thus less pronounced distal dynein accumulation, in vivo data from adult nerves (Dillman et al., 1996a, Dillman et al., 1996b) demonstrate that slow axonal transport persists into adulthood.

### Cytoplasmic Dynein Is Dependent on Kinesin-1 Transport for Localization

Genetic perturbations in model organisms have previously suggested that the localization of dynein in cells is dependent on kinesin-1. For example, *Drosophila Khc* mutants show impaired retrograde transport of mitochondria in axons (Pilling et al., 2006). Similarly, the polar localizations of dynein and kinesin are co-dependent during the establishment of motor-dependent asymmetric mRNA localization early in *Drosophila* oogenesis, where recent work found that posterior localization of dynein could be rescued in *Khc* null oocytes by expression of a KIF5 construct lacking the C-terminal cargo binding tail region (the last 125 amino acids), but not a shorter construct lacking the KLC binding region (Williams et al., 2014). These genetic data are in good agreement with our biochemical data on the DIC interaction with KIF5, where we found an interaction with the stalk region of KIF5, but not the C-terminal tail. In a proof-of-principle experiment, a minimal complex for dynein transport by kinesin was reconstituted from yeast proteins (Roberts et al., 2014), using a truncated dynein heavy chain, Kip2 (a member of the kinesin-7 family), and the yeast orthologs for EB1, CLIP-170 and Lis1. However, this complex is unlikely to play a role in neurons; the closest equivalent to Kip2 is the centromere-associated motor CENP-E, which is specialized to perform chromosome alignment in mitosis and is not known to have a role in axonal transport.

Filamentous fungi are excellent model organisms for the study of microtubule transport as they are both genetically tractable and their long hyphal compartments are axon-like with uniformly orientated plus ends out microtubules (Egan et al., 2012a). In *Aspergillus nidulans*, loss-of-function mutants demonstrate a cooperative role for dynein and dynactin in their plus-end localization to the hyphal tip, localization that is also dependent on the KIF5 ortholog, KINA (Zhang et al., 2003). This kinesin-1-dependent localization of dynein and dynactin in distal hyphae also occurs in *Ustilago maydis* (Lenz et al., 2006). Although not directly addressed in our study, the accumulation of dynactin in axon terminals of both *Drosophila* and mice is also known to be kinesin-1 dependent (Lloyd et al., 2012, Moughamian and Holzbaur, 2012). As a frequent binding partner of dynein, dynactin may also be part of the anterograde transport complex that we have described. In fact, it appears that the basic dynein:kinesin complex may be conserved across species, while additional neuron-specific interactions between DIC and KLC may enhance association to the levels required for a significant accumulation by axonal transport.

There are two mechanisms of targeting dynein to microtubule plus tips: (1) vectorial delivery along microtubules, and (2) direct recruitment to the plus tip from the cytosol. In large polarized cells, these represent two distinct necessities—first, the need to concentrate dynein distally so that, second, this enhanced local concentration can be used to facilitate a plus-end-specific loading mechanism. Creating a distinction between these two mechanisms is important as the molecules that direct kinesin-dependent vectorial movement of dynein in yeast (EB1, CLIP-170, and Lis1) are similar to those that direct the ordered recruitment of dynein to the plus tips of microtubules in mammalian systems (EB1, CLIP-170, and dynactin) (Moughamian et al., 2013, Nirschl et al., 2016). We used low-dose taxol to show that the vectorial movement of dynein in the axon is not dependent on dynamic microtubule plus tips and the direct recruitment pathway. In both fungi and neurons, Lis1 acts as an initiation factor for dynein-driven organelle transport and is required for dynein to leave the microtubule plus tip (Egan et al., 2012b, Lenz et al., 2006, Moughamian et al., 2013, Zhang et al., 2003). Thus, in axons we propose the following sequence of events: (1) the kinesin-1-mediated vectorial delivery of dynein; (2) followed by direct recruitment of dynein to the distal microtubule plus tips by EB1, CLIP-170, and dynactin (Nirschl et al., 2016); and (3) the initiation of retrograde transport aided by Lis1.

In an alternative model based on an overexpression study in DRG neurons, the observation of co-transport of Lis1, DIC1, and TUBB3 led to the proposal that dynein is transported anterogradely in the axon through the Lis1-dependent immobilization of dynein on short “transport” microtubules (Yamada et al., 2008). However, tubulins are known to travel at SCa rather than SCb velocities (Grafstein et al., 1970, Hoffman and Lasek, 1975) and so this model is at odds with in vivo data showing that 95% of dynein is either moving in SCb or with vesicles (Dillman et al., 1996b). Thus, the tubulin co-transport model is unlikely to be sufficient to explain the pronounced distal localization of dynein that we have observed. Instead, the observed co-transport of dynein and tubulin may reflect a role for dynein in the active transport of microtubules along the axon (He et al., 2005).

### The “Kinesin-Limited” Model of Slow Axonal Transport

**Figure 8:**
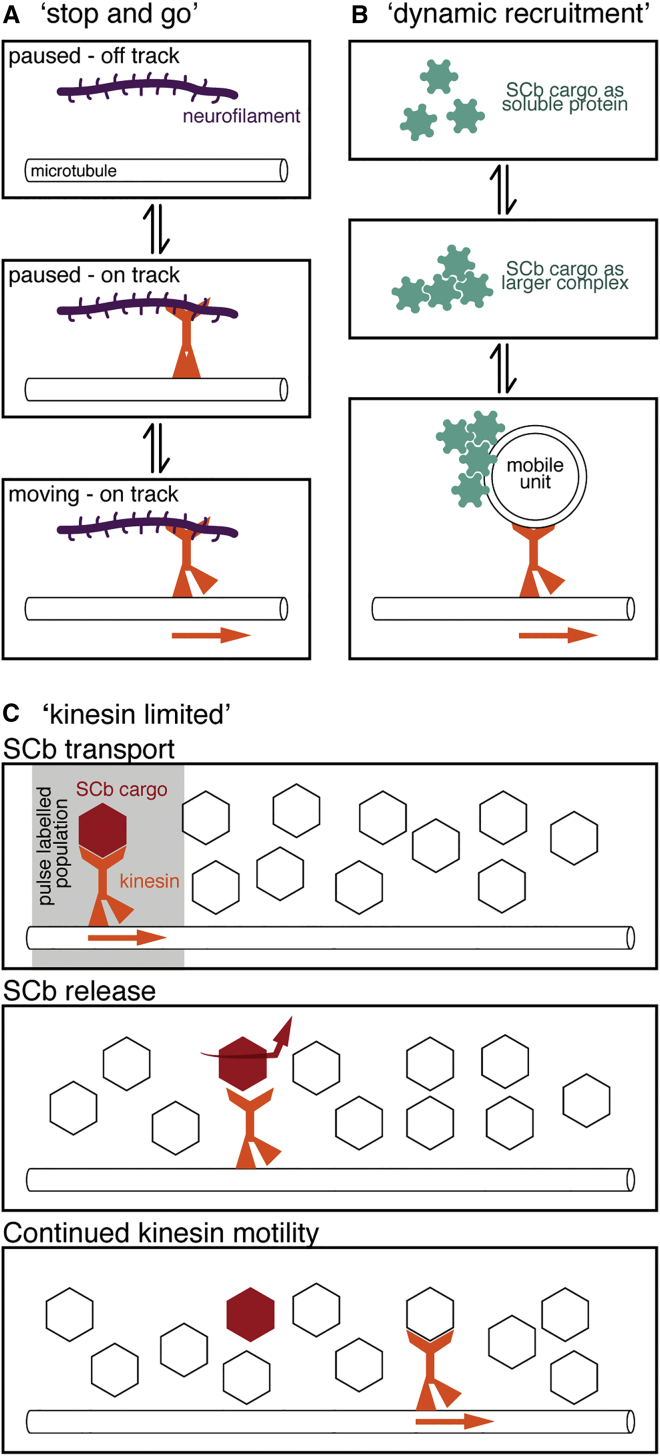
Different Mechanistic Models of Slow Axonal Transport (A) The “stop and go” model describes the transport of neurofilaments in SCa. Through direct association with motors, neurofilaments switch between “off-track” and “on-track” states and between paused and motile states while “on-track” (Brown et al., 2005, Trivedi et al., 2007). (B) The “dynamic recruitment” model describes the transport of some soluble cytosolic proteins moving in SCb, e.g., synapsin I (Tang et al., 2013). Soluble proteins come together to form larger complexes, which stochastically associate with vesicles (the mobile unit) undergoing transport. (C) The “kinesin-limited” model also describes the transport of soluble cytosolic proteins moving in SCb. SCb cargoes such as dynein can directly associate with kinesin for short bursts of motility. By combining a limited ability to hold kinesin in an active state with a relatively low supply of active kinesin motors, slow transport cargoes would move much more slowly relative to kinesin due to the constant binding and release of cargo producing short bursts of motility.

There are currently two models describing slow axonal transport. The “stop and go” model describes the transport of assembled neurofilaments in SCa (Figure 8A) (Brown et al., 2005). Analysis of axonal neurofilament transport shows that neurofilaments cycle between two kinetic states termed “on-track” and “off-track” (Trivedi et al., 2007). On-track neurofilaments display short bouts of movement interrupted by short pauses, while off-track neurofilaments pause for much longer periods. Neurofilaments spend most of their time in the off-track state, producing overall very slow rates of transport. Neurofilaments rely on KIF5A and dynein for transport as assembled polymers, explaining why neurofilament instantaneous velocities are the same as those of fast axonal transport (Uchida et al., 2009, Wagner et al., 2004, Xia et al., 2003), although there are few molecular details about how motors are recruited to neurofilament polymers and why transport is sporadic. The “dynamic recruitment” model of slow axonal transport describes a mechanism in which SCb proteins can traverse the axon via transient formation of larger protein complexes and subsequent recruitment to vesicles for transport (Figure 8B) (Scott et al., 2011, Tang et al., 2013). This model is epitomized by the SCb cargo synapsin I, which has a high affinity for membranes. However, it is difficult to extrapolate this specific behavior to all other cytosolic proteins moving within SCb, as not all proteins have high affinity for membranes.

The coherent transport of numerous SCb proteins in the single peak observed in radiolabelling studies resulted in the hypothesis of a single carrier for SCb (Garner and Lasek, 1982). In truth, there are over 200 (mostly uncharacterized) proteins in SCb and it may be that one transport mechanism will not describe how all proteins are conveyed. The apparent coherence may mask a myriad of different mechanisms due to the limited spatio-temporal resolution possible with this approach.

The neurofilaments of SCa (Uchida et al., 2009) and now dynein in SCb both rely directly on kinesin-1. However, this remains the biggest conundrum: in order to produce such different transport rates, how is the recruitment and activation of kinesin different for slow transport cargos relative to fast vesicle transport? Based on our data demonstrating direct interactions between kinesin and the SCb cargo dynein, we present a third model for slow axonal transport, the “kinesin-limited” model (see below and Figure 8C).

Our model is based on two additional observations. First, a single kinesin has typical run lengths of just a few μm (Friedman and Vale, 1999). Kinesin recruitment to SCb cargo must be less stable than it is to vesicles (as otherwise SCb would have overall transport rates more similar to vesicles), and unstable kinesin recruitment would result in short spontaneous runs followed by dissociation from the microtubule track and disassembly of the transport complex. Second, only a very small percentage of kinesin-1 is observed to be traveling at SCb and SCa velocities in vivo (Elluru et al., 1995). Given the large volumes of protein moved by slow transport relative to fast transport, it is highly likely that there is a limited supply of activated kinesin motors in the axon. If a given SCb cargo loses its bound kinesin, this would naturally lead to long off-track times for SCb cargos and thus an overall net slow transport rate for that cargo. In contrast, the kinesin motor itself would continue to move down the axon in association with a new cargo complex (Figure 8C). This model integrates the common observation of slow transport being kinesin dependent (Terada et al., 2000, Uchida et al., 2009) with the fact that almost all kinesin-1 moves within the fast component of axonal transport (Elluru et al., 1995).

In summary, our kinesin-limited model implies that SCb cargos have a limited ability to recruit and hold kinesin in an active cargo-bound state, within a cytosolic environment with a limited supply of available active kinesin motors (Figure 8). The concept of sporadic transport by “fast” motors underlies all three models for slow axonal transport: “stop and go,” “dynamic recruitment,” and “kinesin limited.” However, our model is distinct from the previous models as it is neither polymer mediated (as for assembled neurofilaments), nor vesicle associated (as for synapsin).

The study of slow axonal transport is challenging due to the slow timescale of the overall transport rates and the indistinct nature of the transport unit for cytosolic cargoes. We have established that the anterograde slow axonal transport of dynein is dependent on direct interactions with kinesin. In doing so, we have established the first set of molecular-level details for a cytosolic slow transport complex, which can now be used to probe the underlying principles of slow axonal transport; in particular, providing insights into the difference between kinesin recruitment for slow compared to fast axonal transport. This work elucidates the dynamic nature of dynein localization in neurons and the mechanism of dynein’s anterograde axonal transport, a critical cellular motor whose function is required up to 1 m from the soma.

## Experimental Procedures

Details of antibodies, cDNA constructs, yeast two-hybrid assay, cell culture, fixed cell imaging, and co-immunoprecipitation can be found in the Supplemental Experimental Procedures.

### Live Cell Imaging

Live cell imaging of hippocampal cultures was performed in Hibernate E low fluorescence medium in a humidified temperature controlled (37°C) live cell chamber, on an Ultraview Vox (PerkinElmer) dual-imaging system mounted on an inverted Nikon Ti microscope with either 100× or 60× apochromat 1.49 NA oil-immersion objectives (Nikon), fully controlled by Volocity software (PerkinElmer). FRAP and slow axonal transport imaging used the spinning-disk confocal with an Ultraview Photokinesis unit for bleaching and a C9100-50 EM-CCD (Hamamatsu) camera. Near-TIRF imaging used the Nikon TIRF system with an EM-CCD C9100-13 camera (Hamamatsu). Image analysis was performed in FIJI (Schindelin et al., 2012) with follow up analysis in R (RStudio). Specifics for each experiment are listed in the Supplemental Experimental Procedures.

### Fluorescence Polarization Measurements

Measurements were performed as previously described (Pernigo et al., 2013) with modifications as listed in Supplemental Experimental Procedures.

### Sucrose Gradient Fractionation

Four mouse brains were homogenized in 16 mL of ice-cold Homogenization buffer (10 mM HEPES [pH 7.4], 0.32 M sucrose, 2 mM EDTA plus protease inhibitors) and subjected to differential centrifugation steps outlined in Supplemental Experimental Procedures. 15%–45% sucrose gradients (15% or 45% sucrose in 10 mM HEPES [pH 7.4], 2 mM EDTA and protease inhibitors) had a 12 mL final volume and 0.5 mL fractions were taken from the top of the gradient for SDS-PAGE and western blotting. Protein concentration and turbidity of fractions was analyzed at 280 and 340 nm, respectively, in a SynergyMx plate reader (BioTek).

For Figure S4A, cytoplasmic dynein and kinesin from the cytosolic pool (S3) was enriched from the brains of adult mice by microtubule affinity and ATP release, followed by sucrose density gradient centrifugation as previously described (Ayloo et al., 2014). Equal fraction volumes were separated by SDS-PAGE and transferred to immobilon-PVDF membrane (Millipore) for western blotting.

### Single Molecule TIRFM Imaging

Flow chambers were formed of a glass slide and silanized (PlusOne Repel Silane, GE Healthcare) coverslip, sandwiched together with adhesive tape and bordered with vacuum grease. Chamber volume was ∼10 μL. The flow chamber was coated with a 1:50 dilution of the monoclonal anti-tubulin antibody (Sigma) then blocked with 5% pluronic F-127 (Sigma). Dual-labeled (AMCA-labeled tubulin seeds with rhodamine tubulin extensions, Cytoskeleton) taxol-stabilized microtubules were flowed into the chamber and allowed to bind the anti-tubulin antibody. The appropriate fraction from the sucrose gradient was supplemented with Mg-ATP (1 mM), bovine serum albumin (1 mg/mL), casein (1 mg/mL), taxol (20 μM), DTT (1 mM), glucose (140 mM), and a glucose catalase/oxidase anti-fade system. Imaging was performed with Ultraview Vox (PerkinElmer) system with 100× apochromat 1.49 NA oil-immersion objective (Nikon). Chambers were imaged at 3–5 frames per second.

### Analysis

All numerical analysis and plotting were performed in R (RStudio), with additional packages dplyr, reshape2, and ggplot2. Statistical comparisons were performed with base R functions as independent (unpaired) t tests, with Bonferroni correction where multiple comparisons were performed (Figures 3G and 3I).

## Author Contributions

A.E.T., G.S., and E.L.F.H. conceived the project and designed experiments. S.P., A.S., R.A.S., and M.P.D. performed and analyzed fluorescence polarization measurements; P.G.-D. performed and analyzed anti-DIC immunofluorescence; A.E.T. performed and analyzed all other experiments. A.E.T. and E.L.F.H. wrote the manuscript. All authors reviewed and edited the manuscript.

## Supplemental Information

Document S1. Supplemental Experimental Procedures and Figures S1–S5

Movie S1. Related to Figure 2. Neurite Outgrowth in a Dynein-GFP Stage 2 Hippocampal Neuron Imaged Overnight Time stamp, hours:minutes. Fluorescence intensity scale as for Figure 2A

Movie S2. Related to Figure 2. Neurite Outgrowth in Dynein-GFP in Stage 2 Hippocampal Neuron Transfected with Constitutively Active K560-Halo and Imaged Overnight Time stamp, hours:minutes. Left: K560-Halo; middle: dynein-GFP; right: K560-Halo (magenta) and dynein-GFP (green) merge.

Movie S3. Related to Figure S2. Neurite Outgrowth in Stage 2 Hippocampal Neuron Transfected with Both K560-Halo and BFP and Imaged Overnight Time stamp, hours:minutes. Left: K560-Halo; middle: BFP; right: K560-Halo (magenta) and BFP (green) merge.

Document S2. Article plus Supplemental Information
