# Supplementary figures and images for: The Dynamic Localization of Cytoplasmic Dynein in Neurons Is Driven by Kinesin-1
Source: Neuron. 2016 Jun 1;90(5):1000–15. doi: 10.1016/j.neuron.2016.04.046 (PMC4893161; doi:10.1016/j.neuron.2016.04.046)

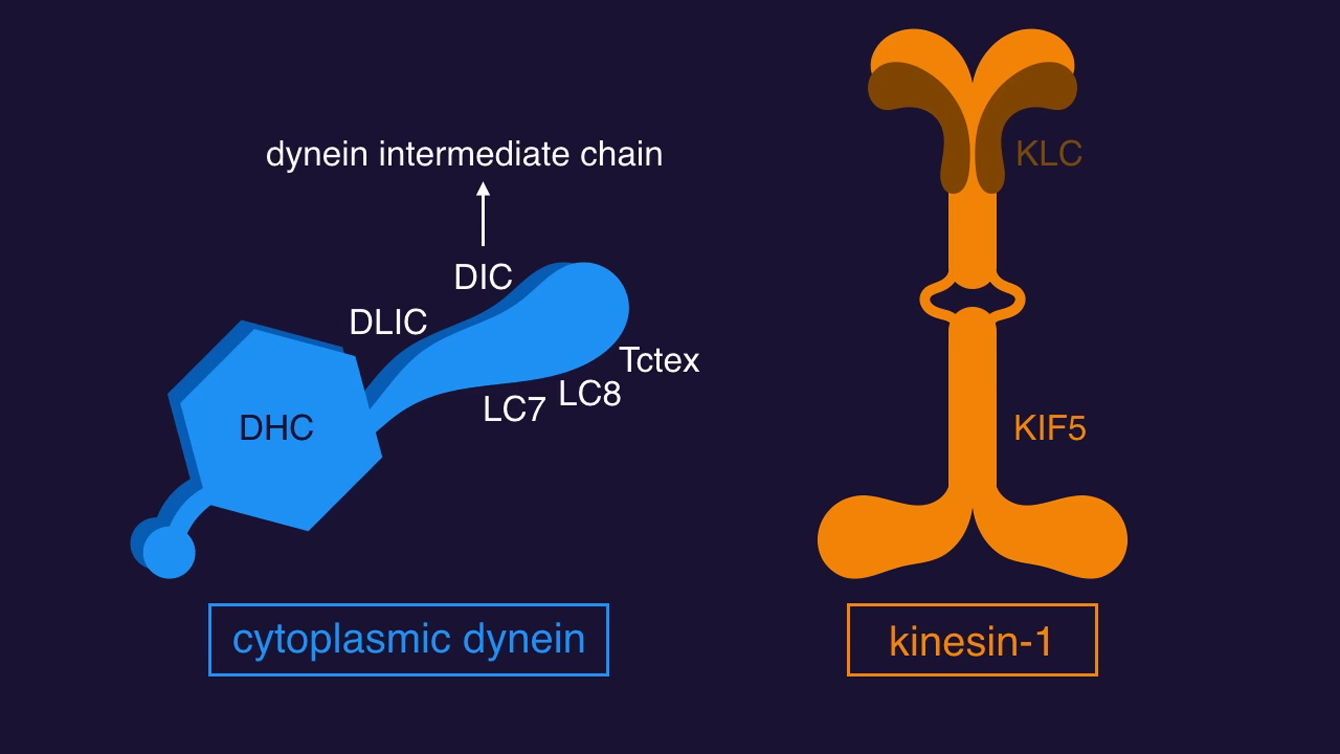

Supplement: Supplementary file 1 [file mmc6.jpg]

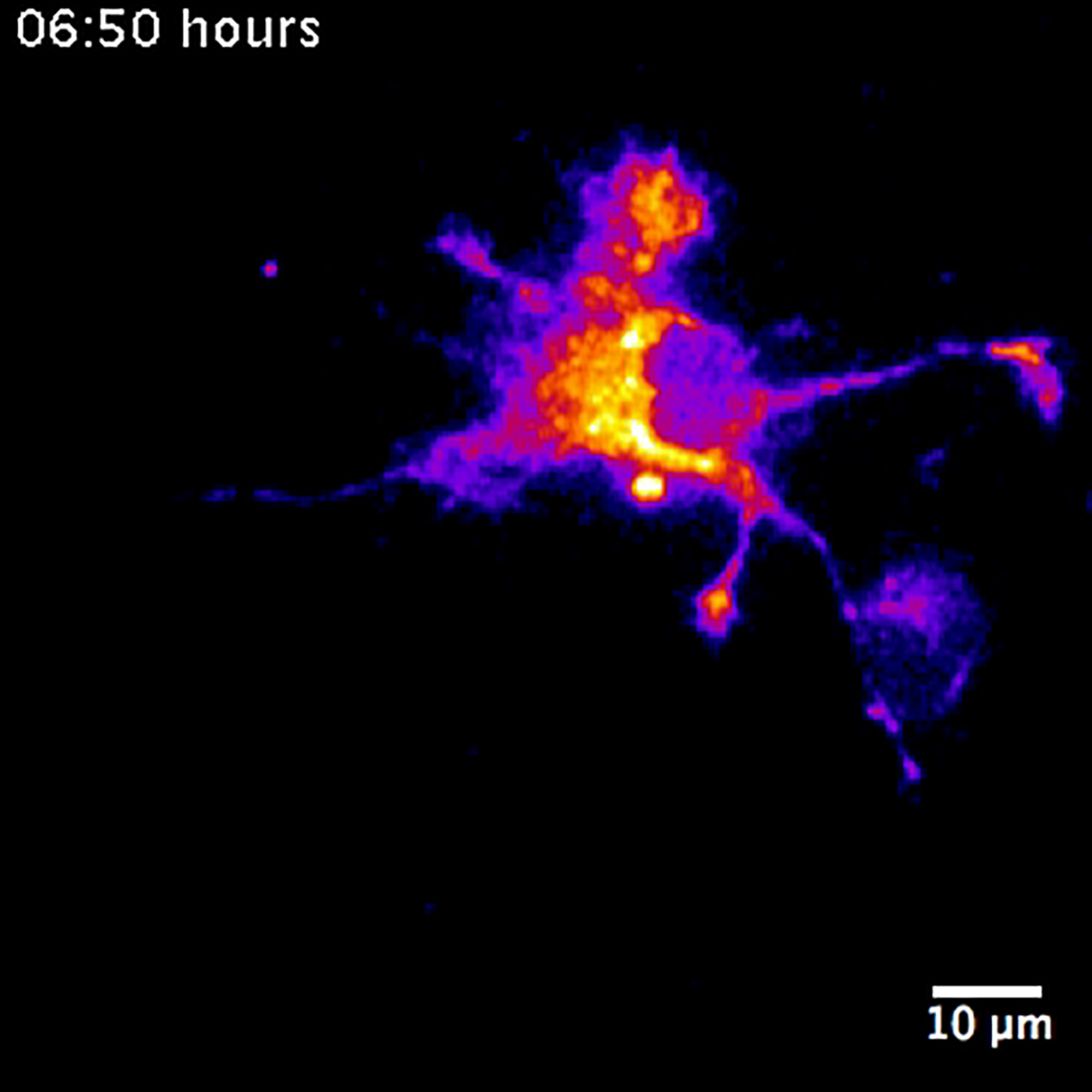

Supplement: Movie S1. Related to Figure 2. Neurite Outgrowth in a Dynein-GFP Stage 2 Hippocampal Neuron Imaged Overnight — Time stamp, hours:minutes. Fluorescence intensity scale as for Figure 2A [file mmc2.jpg]

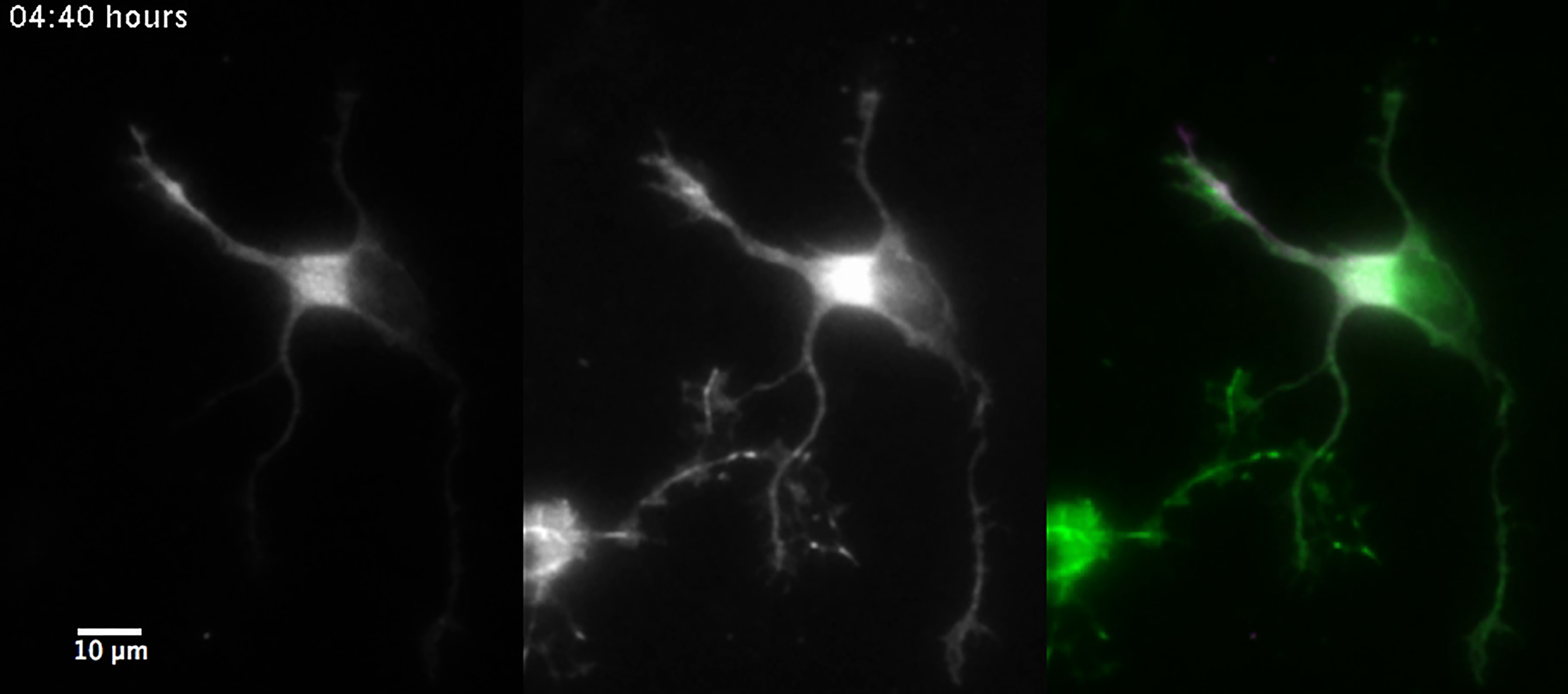

Supplement: Movie S2. Related to Figure 2. Neurite Outgrowth in Dynein-GFP in Stage 2 Hippocampal Neuron Transfected with Constitutively Active K560-Halo and Imaged Overnight — Time stamp, hours:minutes. Left: K560-Halo; middle: dynein-GFP; right: K560-Halo (magenta) and dynein-GFP (green) merge. [file mmc3.jpg]

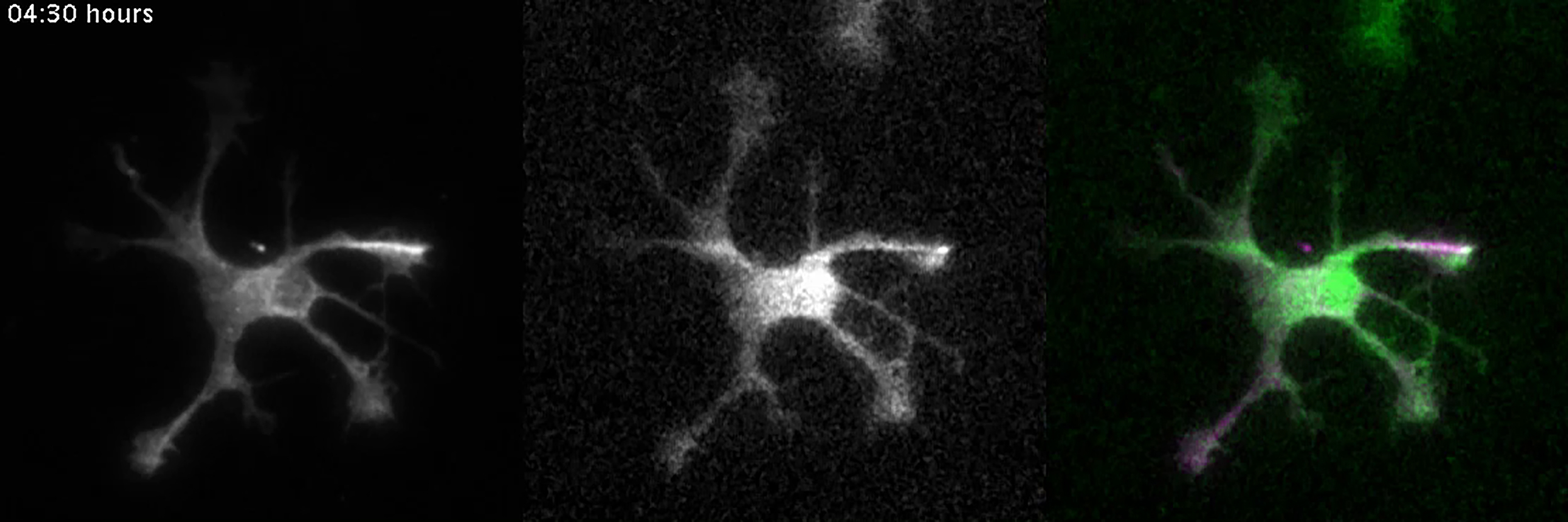

Supplement: Movie S3. Related to Figure S2. Neurite Outgrowth in Stage 2 Hippocampal Neuron Transfected with Both K560-Halo and BFP and Imaged Overnight — Time stamp, hours:minutes. Left: K560-Halo; middle: BFP; right: K560-Halo (magenta) and BFP (green) merge. [file mmc4.jpg]
