# Supplementary material for: The Dynamic Localization of Cytoplasmic Dynein in Neurons Is Driven by Kinesin-1
Source: Neuron. 2016 Jun 1;90(5):1000–15. doi: 10.1016/j.neuron.2016.04.046 (PMC4893161; doi:10.1016/j.neuron.2016.04.046)
Supplement: Document S1. Supplemental Experimental Procedures and Figures S1–S5 [file mmc1.pdf]

Neuron, Volume *90*

Supplemental Information

The Dynamic Localization of Cytoplasmic Dynein in Neurons Is Driven by Kinesin-1

Alison E. Twelvetrees, Stefano Pernigo, Anneri Sanger, Pedro Guedes-Dias, Giampietro Schiavo, Roberto A. Steiner, Mark P. Dodding, and Erika L.F. Holzbaur

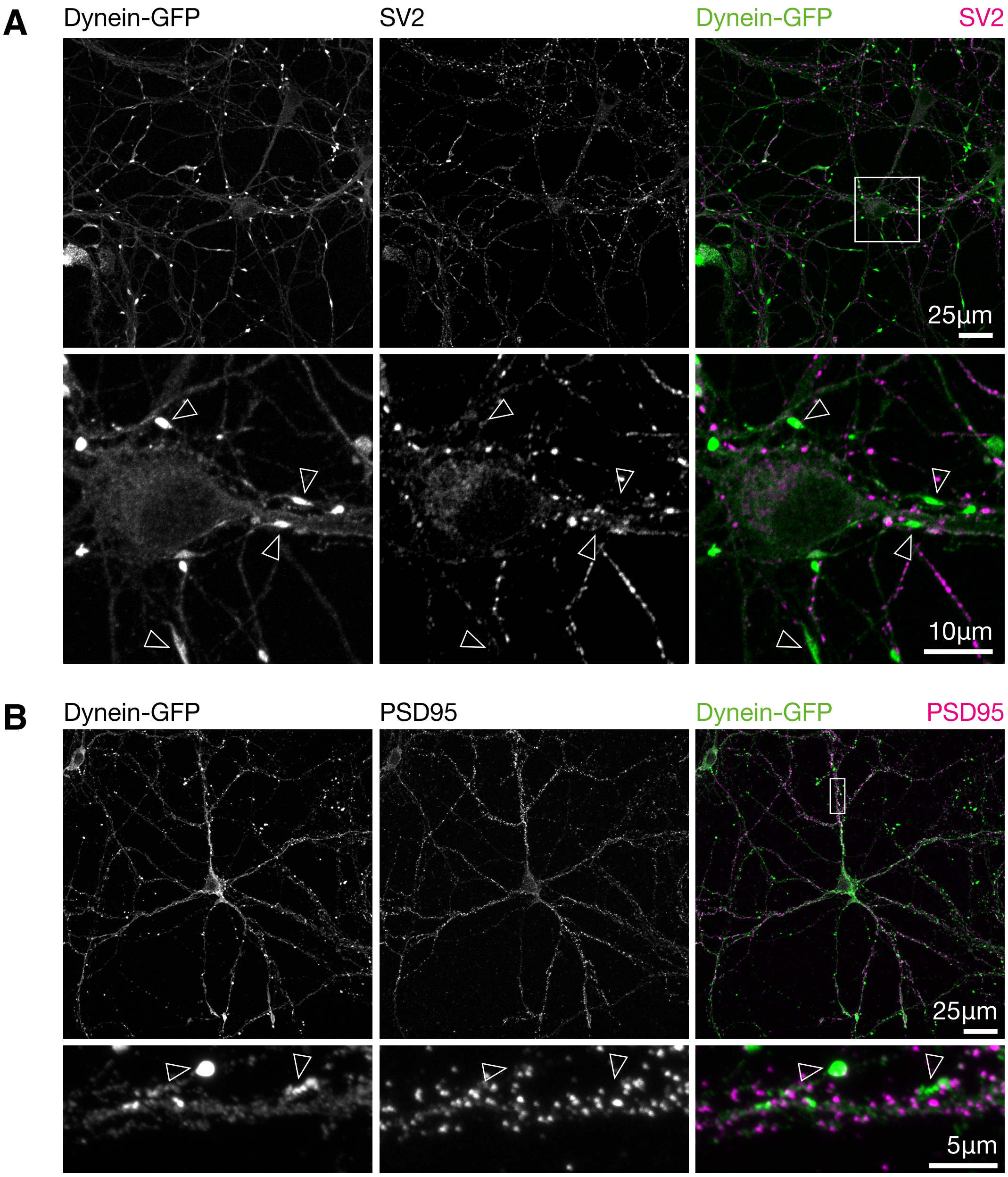

Figure S1. Related to Figure 1.

(A) Dynein-GFP is not enriched at pre-synaptic sites. Immunofluorescence and confocal microscopy of 8 DIV primary hippocampal neurons shows dynein-GFP accumulation does not colocalise with the presynaptic marker SV2 (empty arrowheads).

(B) Dynein-GFP is not enriched at post-synaptic sites. Immunofluorescence and confocal microscopy of mature 21 DIV primary hippocampal neurons shows dynein-GFP axonal foci do not colocalise with the postsynaptic marker PSD-95 (empty arrowheads).

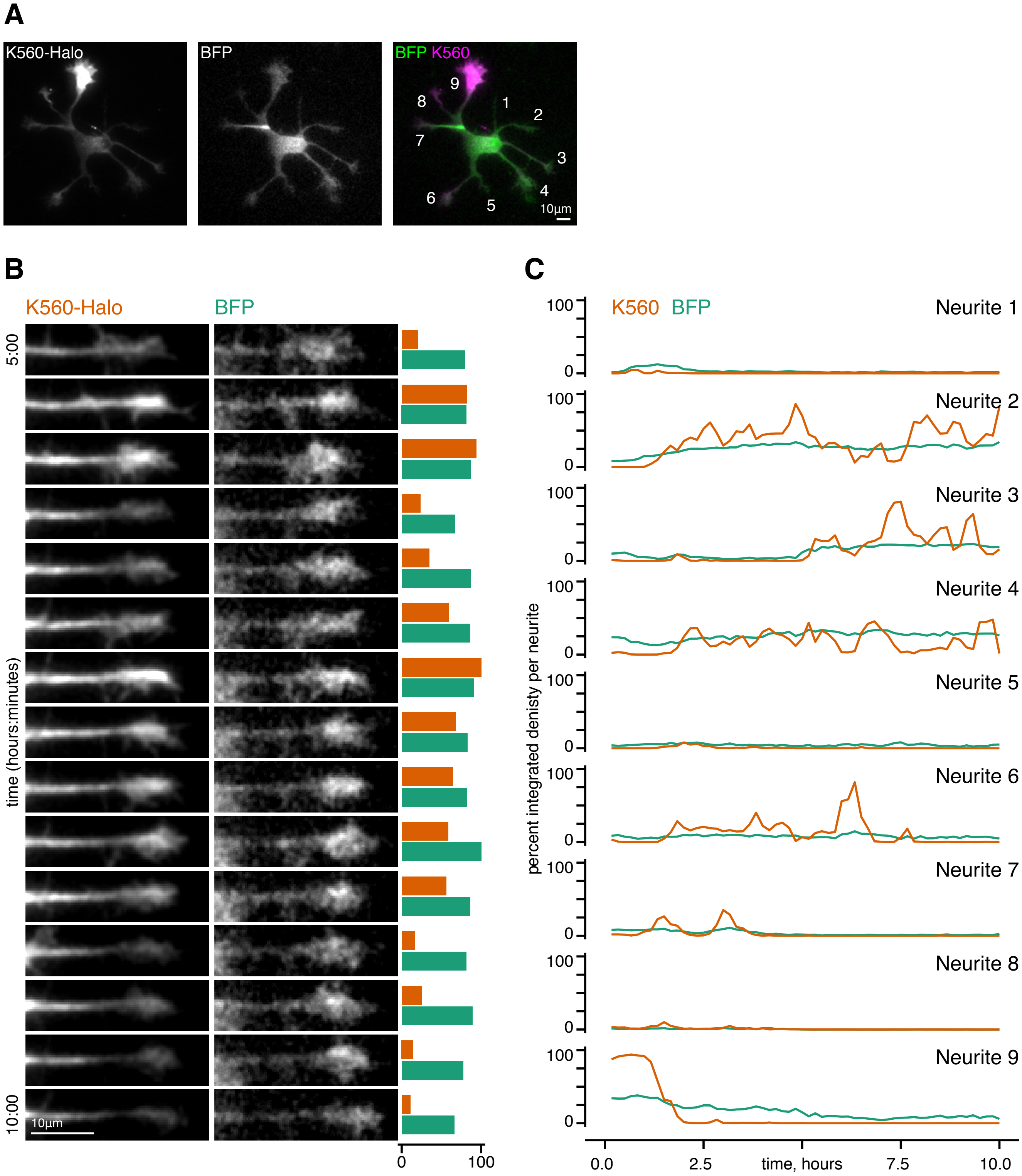

Figure S2. Related to Figure 2.

(A) Dynein-GFP neuron transfected with the cytosolic marker BFP. Maximum projection of all frames in Movie S3 indicating neurite labelling used for quantification in B-C. Scale bar, 10 μm.

(B) The movement of dynein-GFP within developing neurites is not mirrored by BFP. Still images of Neurite 4 from (A) through time. Quantification bars to the right show the relative integrated density of fluorescence for K560-Halo (orange) and BFP (green) within Neurite 4 over time. Scale bar, 10 μm.

(C) The movement of dynein-GFP across developing neurites is not mirrored by BFP. Quantification of the neurite intensity as a percentage of the total in all neurites through time, demonstrating the absence of BFP accumulation in any one neurite relative to other developing neurites labelled in D.

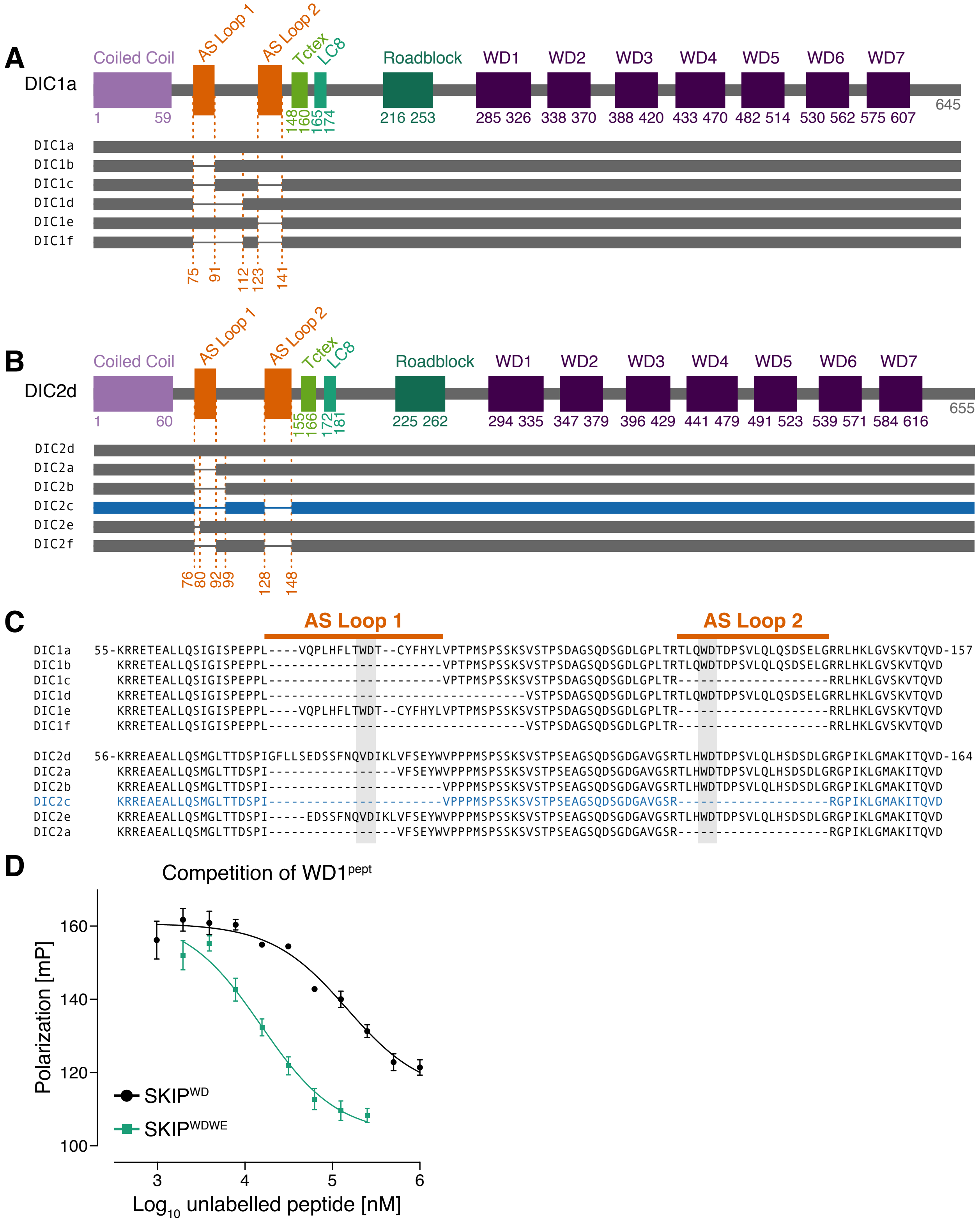

Figure S3. Related to Figure 3.

(A-C) Isoforms of the mouse cytoplasmic dynein intermediate chain, based on data from (Kuta et al., 2010). The splice forms of DIC1 (A) and DIC2 (B) from the genes Dync1i1 and Dync1i2 respectively, relative to the longest isoform of each with key structural elements indicated: coiled coil required for NudE and p150 binding (McKenney et al., 2011; Nyarko et al., 2012); binding sites of the dynein light chains Tctex (Mok et al., 2001), LC8 (Lo et al., 2001) and Roadblock (Susalka et al., 2002); WD repeats 1-7 of the WD40 domain. Sequence alignments of all DIC isoforms centred on the two alternatively spliced regions: AS Loop 1 and AS Loop 2 (C). The location of the two tryptophan motifs within both alternatively spliced regions are highlighted in grey. The ubiquitous DIC isoform, DIC2c, is shown in blue. DIC2c does not contain either AS Loop 1 or 2.

(D) In a fluorescence polarization competition assay, TAMRA-labeled WD1^{pept} bound to KLC1^{TPR} is competed off with both unlabeled SKIP^{WD} (with a single tryptophan motif) or a longer peptide encompassing both motifs (SKIP^{WDWE}). Both SKIP peptides compete off WD1^{pept} in a concentration dependent manner, with the longer and higher affinity SKIP^{WDWE} peptide doing so more efficiently. The sequence of all peptides used is in Supplemental Experimental Procedures. Error bars ± sem, experiments typically done in triplicate.

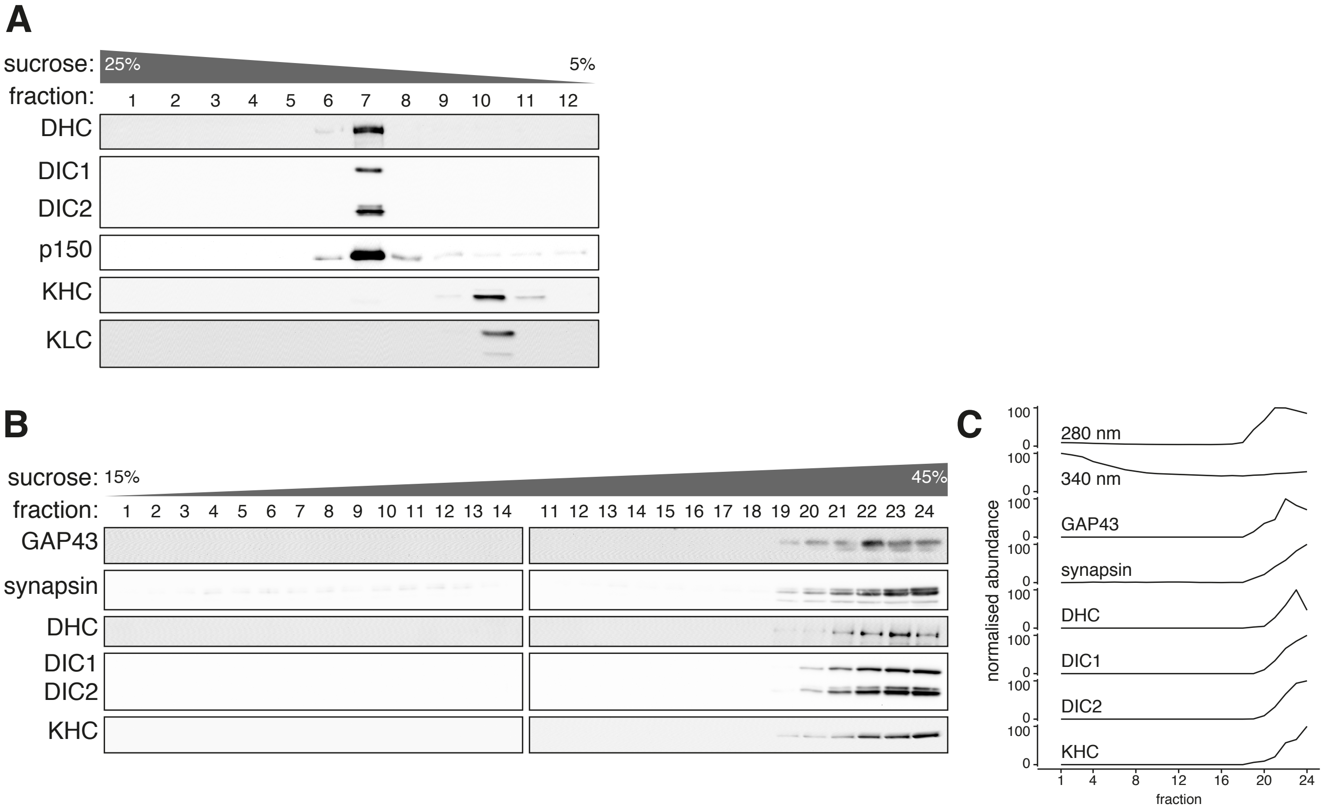

Figure S4. Related to Figure 5.

Biochemical Analysis of endogenous dynein-GFP and kinesin complexes from brain.

(A) Microtubule motors enriched from the cytoplasmic (S3) fraction of brain lysate by their ability to bind microtubules, subsequently separated by sucrose gradient. Dynein and kinesin motors do not co-migrate in this preparation.

(B) Density gradient assay of the resuspended P3 fraction performed in parallel to that in Figure 5D, but in the presence of Triton-X-100 to solubilise vesicles. Protein that was previously floating in less dense fractions of the gradient (see Figure 5D) as part of the vesicle fraction is now collapsed into the high density fractions at the bottom of the gradient.

(C) Quantification of the gradient assay in (A) showing the absence of the vesicular associated protein in fractions 10-19.

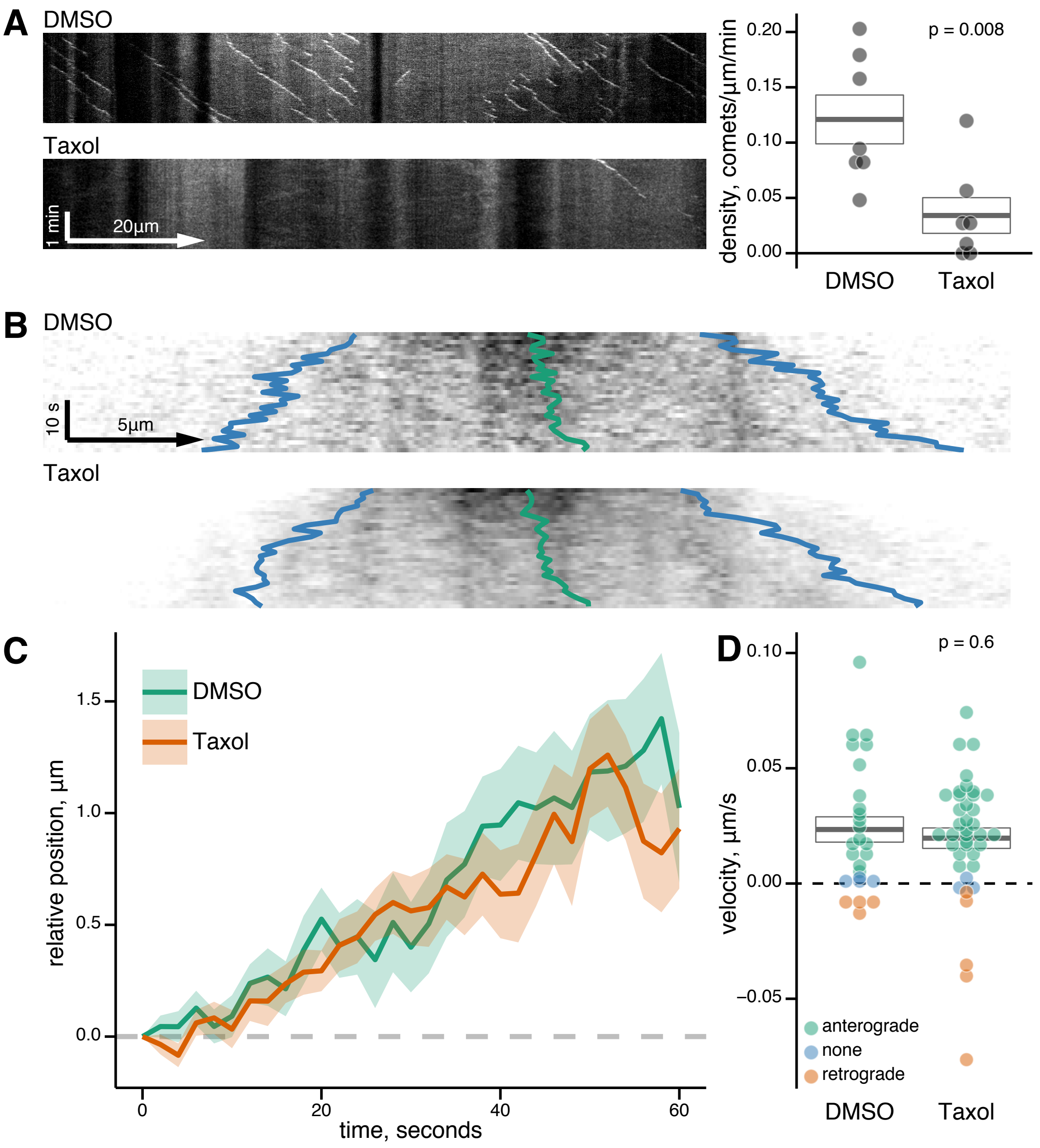

Figure S5. Related to Figure 7.

The slow anterograde transport of dynein in the axon is not dependent on dynamic microtubules.

(A) Taxol treatment reduces the density of EB3 comets in the axon. Example kymographs (left) and quantification (right) of EB3-mCherry comet density in DMSO control and taxol treated axons. Pre-treatment with taxol (200 nM final concentration) for 45-60 minutes prior to imaging reduces the density of EB3 comets in the axon from 0.12 to 0.03 comets/μm/min. Heavy line = mean, box = sem, grey dots = individual axons. Axons were imaged at 1 frame per second for 3 minutes using live cell spinning disk confocal microscopy.

(B) Example slow axonal transport kymographs of DMSO control and taxol treated axons from dynein-GFP hippocampal neurons showing relative positions of anterograde and retrograde intercept (blue) and the calculated midpoint displacement (green) following the bleaching protocol used to analyse slow axonal transport (see Figure 6).

(C) Quantification of the midpoint displacement shows that taxol treatment has no affect on the anterograde bias of dynein-GFP transport in the axon. The mean relative position of the midpoint with time for DMSO and taxol treated axons: n = 26 and 39 axons respectively from 3 independent primary cultures; solid lines, mean; ribbons, ± sem.

(D) The mean velocity of dynein displacement in the axon is unaffected by taxol treatment. A linear regression was fitted to each midpoint displacement to find the velocity of displacement. The mean velocity (heavy line) ± sem (box) is shown. Overlaid spots are the velocities for each measured kymograph with colour indicating the overall direction of the midpoint displacement. Taxol treatment causes no changes to the mean velocity of anterograde displacement. DMSO mean velocity 0.023±0.005 μm/s; Taxol mean velocity 0.020±0.004 μm/s. p value calculated by a two-sample t-test.

Movie S1. Related to Figure 2.

Neurite outgrowth in a dynein-GFP stage 2 hippocampal neuron imaged overnight. Time stamp, hours:minutes. Fluorescence intensity scale as for Figure 2A.

Movie S2. Related to Figure 2.

Neurite outgrowth in dynein-GFP in stage 2 hippocampal neuron transfected with constitutively active K560-Halo and imaged overnight. Time stamp, hours:minutes. Left panel, K560-Halo; Middle panel, dynein-GFP; Right panel, K560-Halo (magenta) and dynein-GFP (green) merge.

Movie S3. Related to Figure S2.

Neurite outgrowth in stage 2 hippocampal neuron transfected with both K560-Halo and BFP and imaged overnight. Time stamp, hours:minutes. Left panel, K560-Halo; Middle panel, BFP; Right panel, K560-Halo (magenta) and BFP (green) merge.

Supplemental Experimental Procedures

Antibodies

Primary antibodies used: chicken anti-GFP (Aves Labs, GFP-1020); mouse anti-mCherry (Living Colors, 632543); mouse anti-FLAG(M2) (Sigma, F1804); rabbit anti-Tau (Abcam); mouse anti-SV2 (recognises all three SV2 isoforms); mouse anti-DIC (Millipore, MAB1618); rabbit anti-GAP43 (Chemicon, MAB 347); anti-PSD95 (Millipore, MAB1596); rabbit anti-synapsin; rabbit anti-DHC (Santa Cruz); mouse anti-p150 (BD biosciences, 610474); mouse anti-KHC/KIF5 (Millipore, MAB1614); mouse anti-KLC (Millipore, MAB1616); rabbit anti-KLC2 (Abcam, ab116702).
Fluorophore conjugated secondary antibodies were Cy2 conjugated goat anti-chicken (Jackson) or goat anti-chicken Alexa 488 (ThermoFisher); and goat anti-rabbit Alexa 594 (Invitrogen), anti-mouse Alexa 594 (Invitrogen) or goat anti-mouse Alexa 555 (ThermoFisher). HRP conjugated secondary antibodies were from Jackson.

cDNA Constructs

All kinesin constructs used have been previously described (Fu and Holzbaur, 2013; Ligon et al., 2004; Twelvetrees et al., 2010). The DIC1a construct was originally a gift from Richard Vallee. mCherry tagged DIC1a was made by replacing the EGFP tag in pEGFP-N1 with mCherry. Site-directed mutagenesis was carried out with Agilent (Stratagene) OC lightening Multi Side-directed Mutagenesis Kit following manufacturers instructions. Every base of mutant constructs was confirmed by sequencing.

Yeast two-hybrid assay

Previous studies have demonstrated a direct interaction between DIC1 and either KLC1 or KLC2 (Ligon et al., 2004). In a yeast two-hybrid assay to screen a random-primed human fetal brain cDNA library for further interactors of DIC1 as described in (Perlson et al., 2013), we identified a cDNA encoding residues 389-637 of KIF5A as a possible interaction partner. Interactions between DIC1A and KIF5A, KIF5B, and KIF5C were verified by co-immunoprecipitation experiments, while the binding domain within KIF5C was identified by comparing co-precipitation of mCherry-DIC1 with KIF5C head, stalk, and tail constructs.

Primary hippocampal neuronal culture

Hippocampal neurons were isolated from E15.5 dynein-GFP mice and cultured as previously described (Zhang et al., 2013). For microfluidic chambers (RD900, Xona Microfluidics) 300,000 neurons were seeded into each chamber and maintained by exchange of 30% of the maintenance media every day.

Immunocytochemistry and confocal imaging

Hippocampal neurons were fixed and stained as previously described (Twelvetrees et al., 2010). Figures 1B&C and S1A&B were imaged with Zeiss LSM 710 with a Plan-Apochromat 63x/1.40 NA oil immersion objective and are maximum projections of z-stacks taken at the recommended sampling rate with pinhole set to 1 Airy unit (planes that included just neuronal soma were excluded from the projection). Figures 1D-E were imaged using UltraVIEW VoX spinning-disk confocal microscope (PerkinElmer) with an Apochromat 100X, 1.49 NA oil immersion objective (Nikon). Digital images were acquired with C9100-50 EM-CCD camera (Hamamatsu). In order to show axon/dendritic labeling alongside bright puncta, images were contrast enhanced for presentation using FIJI/ImageJ. Tiling of overlapping fields of view in Figure 1D-E was done in ImageJ (D) or Photoshop (E). Pearson's correlation coefficient was calculated using Velocity software. Note that all dynein-GFP fixed cells were stained with anti-GFP antibodies to boost the GFP signal for confocal imaging.

Live cell imaging

FRAP

Photobleaching of dynein-GFP (Figure 1F) was performed with 488 nm laser at 100% power for 25 cycles using the 100X objective. Pre-bleach frames were acquired at 2 frames per second (2 fps), and post bleach frames at 2 frames per minute (fpm).

near-TIRF real time imaging

Axons were imaged with 100X objective and near-TIRF illumination with 488 nm laser. Frame rate was 3 fps for Figure 1G. Bleaching in Figure 1H was performed with the Ultraview Photokinesis device before switching to TIRF imaging mode and imaging recovery at 5 fps post bleaching.

Overnight live cell imaging

Cells were imaged with 60X objective with near-TIRF illumination to eliminate excessive fluorescence signal coming from out of focus soma. Acquisition was run overnight with frames acquired at 1 frame every ten minutes, with stage position controlled by Velocity software. For quantification, a threshold was applied to a maximum projection to define neurite regions of interest (ROI). These ROI were overlaid onto the movie and integrated density of each neurite was measured over time.

Slow axonal transport imaging and drug treatments

Axons were imaged with a 100X objective (Nikon). Acquisition speed was 1 frame every two seconds.
For Nocodazole and Taxol drug treatments, 33 µM and 200 nM respective final concentrations were added to each well of the microfluidic chamber in imaging media (Hibernate E Low Fluorescence media) 45-60 minutes prior to imaging, with hydrostatic pressure across the microgrooves of the microfluidic device to ensure drugs reached the axons within the grooves.
Peptides were supplied by GenScript, and sequences are VCFYLLLQTWVFPYTHDLH and LVQPLHFLTWDTCYFHYLV for Control and DIC1a-WD1 peptides respectively. For peptide treatments, 500 ng of peptide was complexed with Chariot reagent (Active Motif) following the manufacturer's instructions then applied to the chamber, immediately followed by imaging media. Peptide treated chambers were incubated for 60 minutes prior to imaging and peptides remained in the imaging media throughout the experiment.
Every suitable isolated axon within the chamber grooves was imaged. Axons without sufficient signal:noise to perform analysis were later discarded (see following). Kymographs of imaged axons were generated in FIJI (Schindelin et al., 2012): axons were straightened along a line the same width as the axon; straightened axons were re-sliced through the t-stack at 1 pixel intervals; 1 pixel kymographs were combined into the final kymograph with an average projection. Kymographs were analyzed with R (RStudio) using the following additional packages: TIFF kymograph images were imported into R using tiff::readTIFF (Urbanek, 2013); rolling average for each time point was performed with zoo::rollapply (Zeileis and Grothendieck, 2005); proximal and distal intercepts at half maximal intensity were calculated with the "approx" function; linear models were fitted with "lm"; additional data handling and plotting used the dplyr (Wickham and Francois, 2015) and ggplot2 libraries respectively (Wickham, 2009). In order to obtain proximal and distal intercepts, the half maximal value has to be above background fluorescence in the axon; of the 31 frames analyzed for each movie, intercepts had to be calculated for at least 30 frames or the data was excluded.

Co-Immunoprecipitation

COS7 cells were passaged 24 hours prior to transfection with Fugene6 (Roche) according to the manufacturer's instructions, and harvested 16-24 hrs after transfection. One 10 cm plate of cells per condition was washed in PBS, transferred to a microcentrifuge tube and lysed in 1ml IP buffer (25 mM HEPES pH 7.4, 150 mM NaCl, 0.5% NP40, 0.5% Triton plus and protease inhibitor cocktail (Roche)) for ten minutes on ice. Cells lysate was harvested by centrifugation at 13,000 g for 10 min at 4˚C. 1 µg of antibody was added to lysate and incubated for 45 minutes at 4˚C with rotation, followed by a further 15

minutes with the addition of 15 µl of washed pre-equilibrated Protein-G Dynabeads. Beads were washed three times in IP buffer and then boiled in 50 µl 1X SDS-loading buffer. Samples were separated by SDS-PAGE and transferred to immobilon-PVDF membrane (Millipore) for western blotting. Primary antibodies listed above. HRP conjugated secondary antibodies were from Jackson. Blots were visualized by detection of enhanced chemiluminescence (SuperSignal West Pico Chemiluminescent Substrate, Thermo Scientific) with a G:Box Chemi system controlled by GeneSys software (Syngene). Image analysis was performed in FIJI (Schindelin et al., 2012).

Fluorescence polarization measurements

N-terminal carboxytetramethylrhodamine (TAMRA) conjugated and unlabeled peptides were supplied by Bio-Synthesis Inc (Lewisville, TX, USA). Sequences are (WD1^{pept}: TAMRA-LHFLTWDTCYF, WD2^{pept}: TAMRA-TRTLQWDTDPS, SKIP^{WD}: STNLEWDDSAI, SKIP^{WDWE}: STNLEWDDSAIAPSSEDYDFGDVFPAVPSVPSTDWEDGDL). The TPR domain of KLC2 lacking the first helix of its first TPR repeat (KLC2^{TPR}, amino acids V218 to K480) was amplified from a mouse template and subcloned into the NdeI/XhoI sites of the pET28a vector (Novagen) to enable its expression with a thrombin-cleavable N-terminal hexa-histidine tag. All fluorescence polarization (FP) measurements were carried out on a BMG Labtech PolarStar Omega plate reader at 20 °C by incubating 300 nM TAMRA-labeled peptides with His6-tagged KLC2^{TPR} at increasing concentrations in 25 mM Hepes pH 7.5, 150 mM NaCl, 5 mM β-mercaptoethanol. Estimation of K_D's assumed a one-site specific-binding model. For competition experiments a mixture of TAMRA-DIC^{WD1} and KLC2^{TPR} at 300 nM and 25 µM, respectively (corresponding to approximately 70% saturation), were incubated with increasing concentrations of unlabeled SKIP^{WD} and SKIP^{WDWE} peptides. Concentration-dependent decrease in FP signal was fitted to sigmoidal curve. Analyses were performed using the Prism package (GraphPad Software Inc., San Diego CA, USA).

Sucrose gradient fractionation

Four mouse brains were homogenized in 16 ml of ice cold Homogenization buffer (10 mM HEPES pH 7.4, 0.32 M sucrose, 2 mM EDTA plus protease inhibitors) then centrifuged at 1000 g for 15 min at 4˚C to form supernatant 'S1' and pellet 'P1'. S1 was centrifuged at 10,000g for 20 min at 4˚C with an F21S-8x50y rotor in Sorval RC6+ centrifuge to form 'S2' and 'P2'. Supernatant S2 was centrifuged at ~100,000 x g for 60 min at 4˚C in Beckman L7 Ultracentrifuge with 70.1 Ti rotor to form 'S3' and 'P3'. P3 was resuspended in 0.6 ml of Homogenization buffer with gentle strokes of a glass-Teflon homogenizer. Samples of S1-P3 were taken at each stage and protein content measured by BCA assay and normalized to 1 mg/ml with 10 µg loaded on gels. P3 was then adjusted to 45% sucrose, and Triton-X-100 added to a final concentration of 1% to half of the sample. P3 fractions with and without Triton were bottom loaded onto 15-45% sucrose gradients (15% or 45% sucrose in 10 mM HEPES pH 7.4, 2 mM EDTA and protease inhibitors) with a 12 ml final volume and centrifuged at 160,000 x g for 16 hr at 4˚C in Beckman L7 Ultracentrifuge with SW41 swinging bucket rotor. 0.5 ml fractions were taken from the top of the gradient. Protein concentration and turbidity of fractions was analyzed at 280 and 340 nm respectively in a SynergyMx plate reader (BioTek). Equal fraction volumes were separated by SDS-PAGE and transferred to immobilon-PVDF membrane (Millipore) for western blotting. Blots were visualized by detection of enhanced chemiluminescence (SuperSignal West Pico Chemiluminescent Substrate, Thermo Scientific) with a G:Box Chemi system controlled by GeneSys software (Syngene). Image analysis was performed in FIJI (Schindelin et al., 2012).

Supplemental References

Fu, M.-M., Holzbaur, E.L.F., 2013. JIP1 regulates the directionality of APP axonal transport by coordinating kinesin and dynein motors. J Cell Biol 202, 495–508. doi:10.1083/jcb.201302078

Kuta, A., Deng, W., El-Kadi, A.M., Banks, G.T., Hafezparast, M., Pfister, K., Fisher, E.M.C., 2010. Mouse Cytoplasmic Dynein Intermediate Chains: Identification of New Isoforms, Alternative Splicing and Tissue Distribution of Transcripts. PLoS ONE 5, e11682. doi:10.1371/journal.pone.0011682

Ligon, L.A., Tokito, M.K., Finklestein, J.M., Grossman, F.E., Holzbaur, E.L.F., 2004. A direct interaction between cytoplasmic dynein and kinesin I may coordinate motor activity. J Biol Chem 279, 19201–19208. doi:10.1074/jbc.M313472200

Lo, K.W., Naisbitt, S., Fan, J.S., Sheng, M., Zhang, M., 2001. The 8-kDa dynein light chain binds to its targets via a conserved (K/R)XTQT motif. J Biol Chem 276, 14059–14066. doi:10.1074/jbc.M010320200

McKenney, R.J., Weil, S.J., Scherer, J., Vallee, R.B., 2011. Mutually exclusive cytoplasmic dynein regulation by NudE-Lis1 and dynactin. J Biol Chem 286, 39615–39622. doi:10.1074/jbc.M111.289017

Mok, Y.K., Lo, K.W., Zhang, M., 2001. Structure of Tctex-1 and its interaction with cytoplasmic dynein intermediate chain. J Biol Chem 276, 14067–14074. doi:10.1074/jbc.M011358200

Nyarko, A., Song, Y., Barbar, E., 2012. Intrinsic disorder in dynein intermediate chain modulates its interactions with NudE and dynactin. J Biol Chem. doi:10.1074/jbc.M112.376038

Perlson, E., Hendricks, A.G., Lazarus, J.E., Ben-Yaakov, K., Gradus, T., Tokito, M.K., Holzbaur, E.L.F., 2013. Dynein Interacts with the Neural Cell Adhesion Molecule (NCAM180) to Tether Dynamic Microtubules and Maintain Synaptic Density in Cortical Neurons. J Biol Chem. doi:10.1074/jbc.M113.465088

Schindelin, J., Arganda-Carreras, I., Frise, E., Kaynig, V., Longair, M., Pietzsch, T., Preibisch, S., Rueden, C., Saalfeld, S., Schmid, B., Tinevez, J.-Y., White, D.J., Hartenstein, V., Eliceiri, K., Tomancak, P., Cardona, A., 2012. Fiji: an open-source platform for biological-image analysis. Nat Methods 9, 676–682. doi:doi:10.1038/nmeth.2019

Susalka, S.J.S., Nikulina, K.K., Salata, M.W.M., Vaughan, P.S.P., King, S.M., Vaughan, K.T., Pfister, K., 2002. The roadblock light chain binds a novel region of the cytoplasmic Dynein intermediate chain. J Biol Chem 277, 32939–32946. doi:10.1074/jbc.M205510200

Twelvetrees, A.E., Yuen, E.Y., Arancibia-Carcamo, I.L., Macaskill, A.F., Rostaing, P.P., Lumb, M.J., Humbert, S.S., Triller, A.A., Saudou, F., Yan, Z.Z., Kittler, J.T., 2010. Delivery of GABAARs to synapses is mediated by HAP1-KIF5 and disrupted by mutant huntingtin. Neuron 65, 53–65. doi:10.1016/j.neuron.2009.12.007

Urbanek, S., 2013. tiff: Read and write TIFF images.

Wickham, H., 2009. ggplot2: elegant graphics for data analysis.

Wickham, H., Francois, R., 2015. dplyr: A grammar of data manipulation. R package version 04 02.

Zeileis, A., Grothendieck, G., 2005. zoo: S3Infrastructure for Regular and Irregular Time Series. J. Stat. Soft. 14. doi:10.18637/jss.v014.i06

Zhang, J., Twelvetrees, A.E., Lazarus, J.E., Blasier, K.R., Yao, X., Inamdar, N.A., Holzbaur, E.L.F., Pfister, K., Xiang, X., 2013. Establishing a novel knock-in mouse line for studying neuronal cytoplasmic dynein under normal and pathologic conditions. Cytoskeleton (Hoboken, NJ) 70, 215–227. doi:10.1002/cm.21102
